# Supplementary figures and images for: Soil metabolomics and bacterial functional traits revealed the responses of rhizosphere soil bacterial community to long-term continuous cropping of Tibetan barley
Source: PeerJ. 2022 Apr 7;10:e13254. doi: 10.7717/peerj.13254 (PMC8995024; doi:10.7717/peerj.13254)

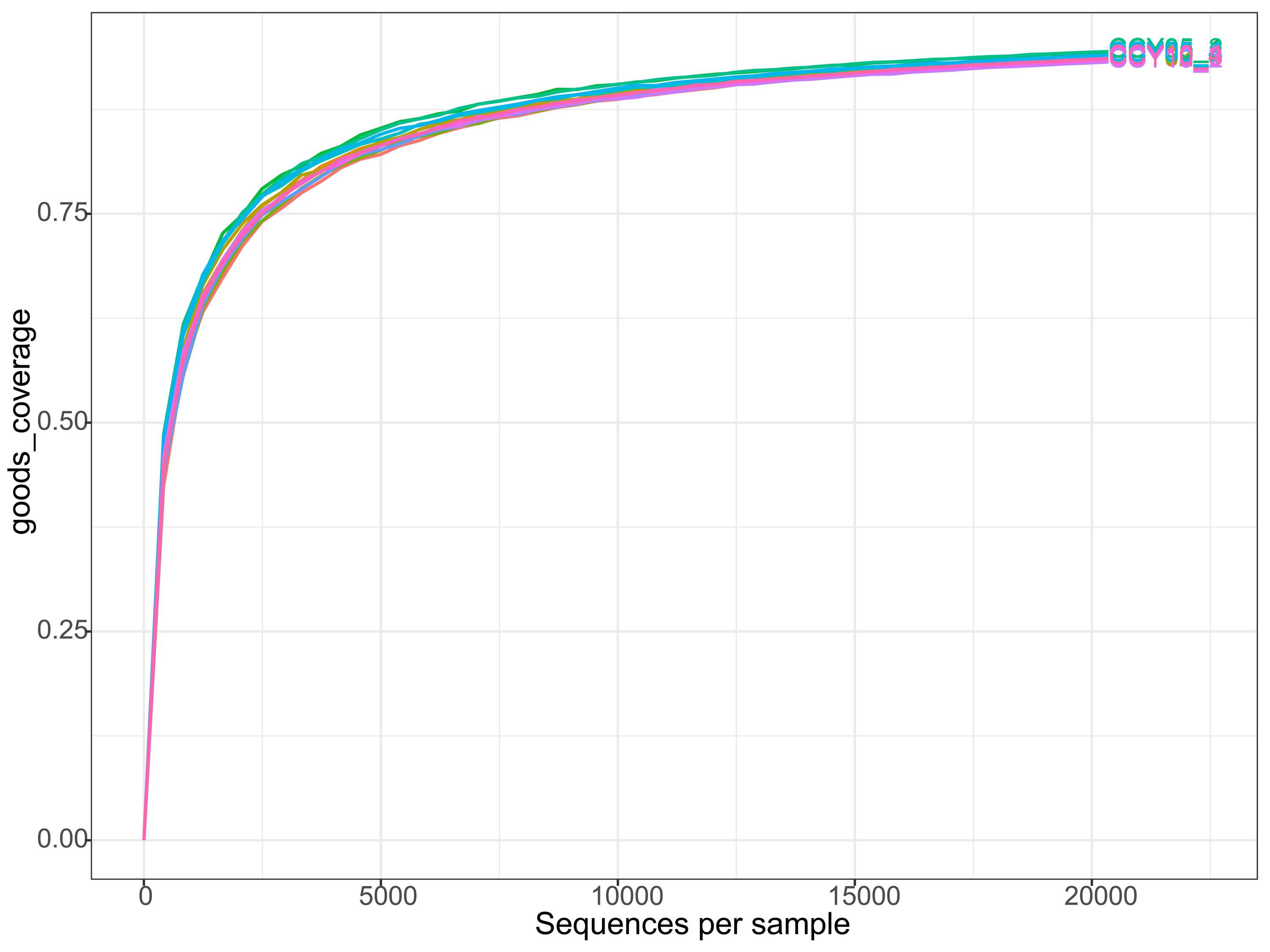

Supplement: Figure S1 [file peerj-10-13254-s001.png]

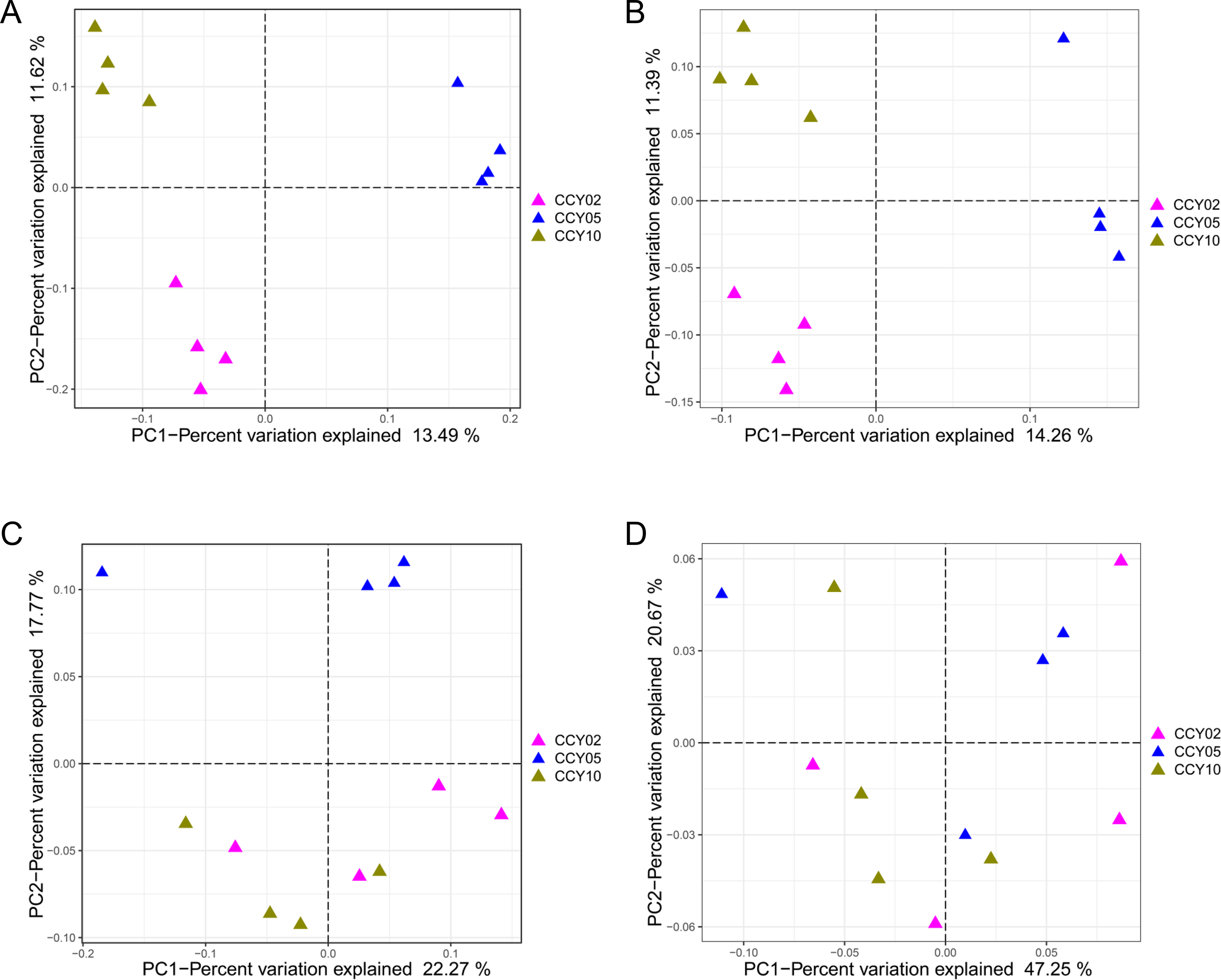

Supplement: Figure S2 [file peerj-10-13254-s002.png]

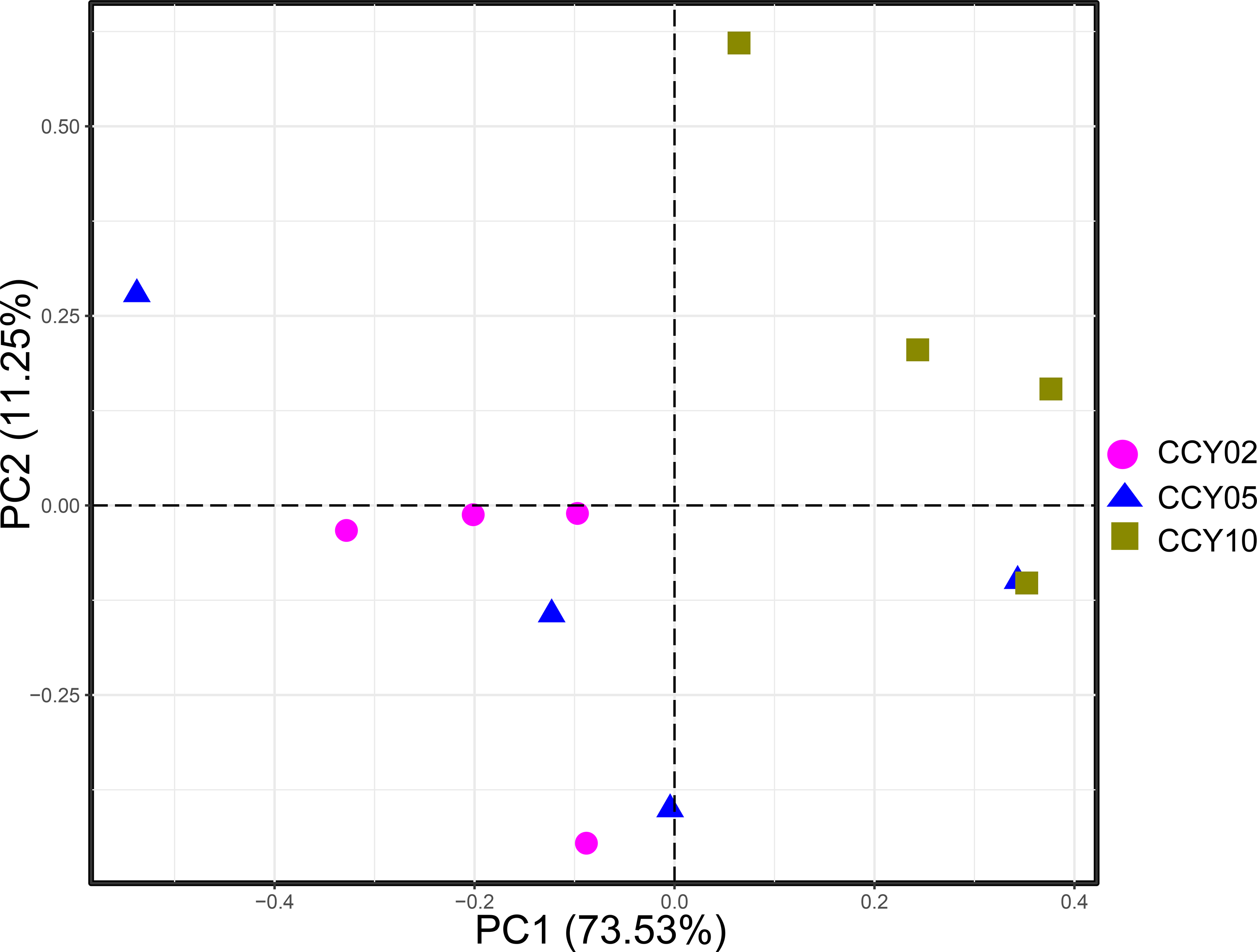

Supplement: Figure S3 [file peerj-10-13254-s003.png]

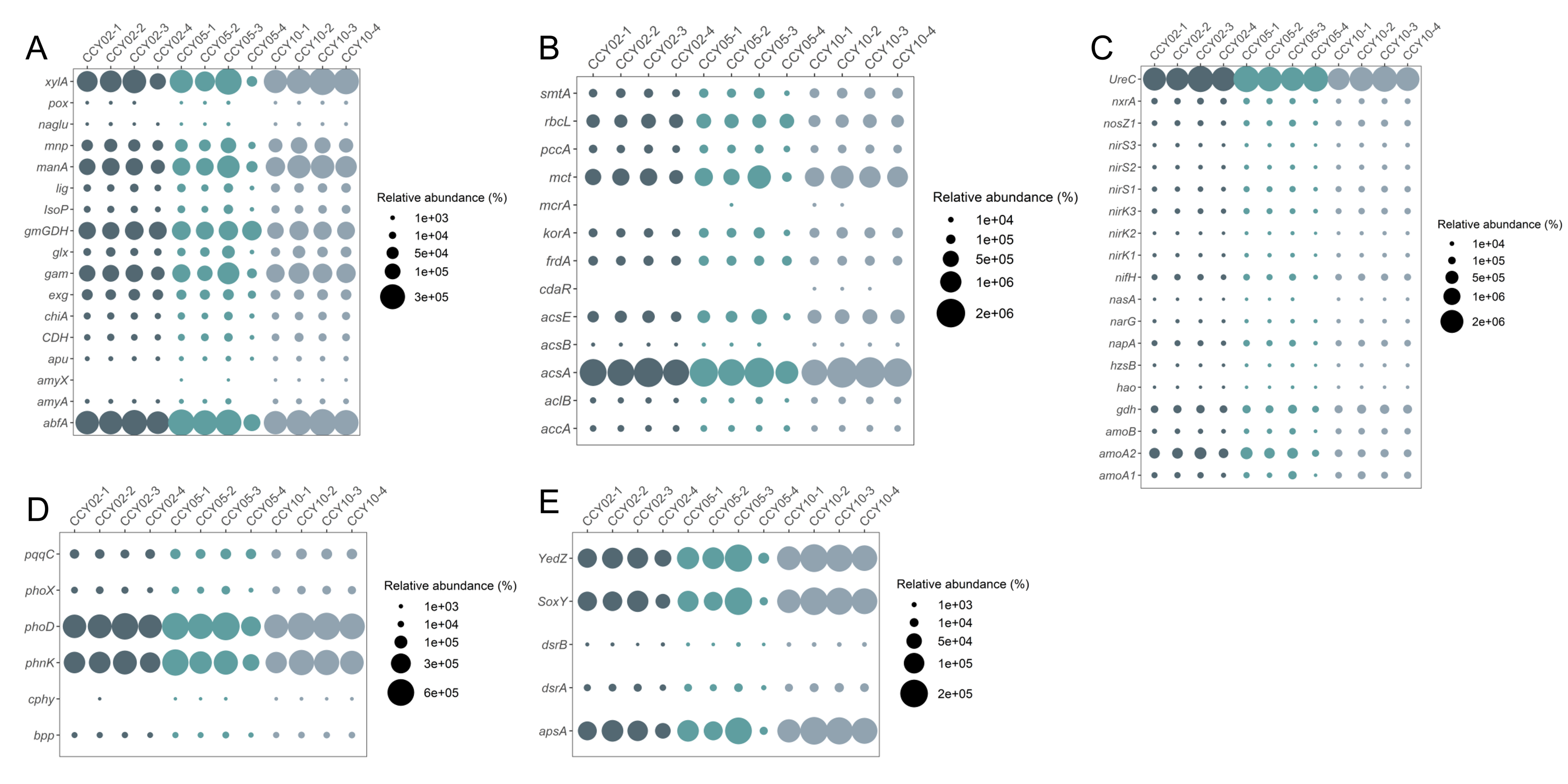

Supplement: Figure S4 [file peerj-10-13254-s004.png]

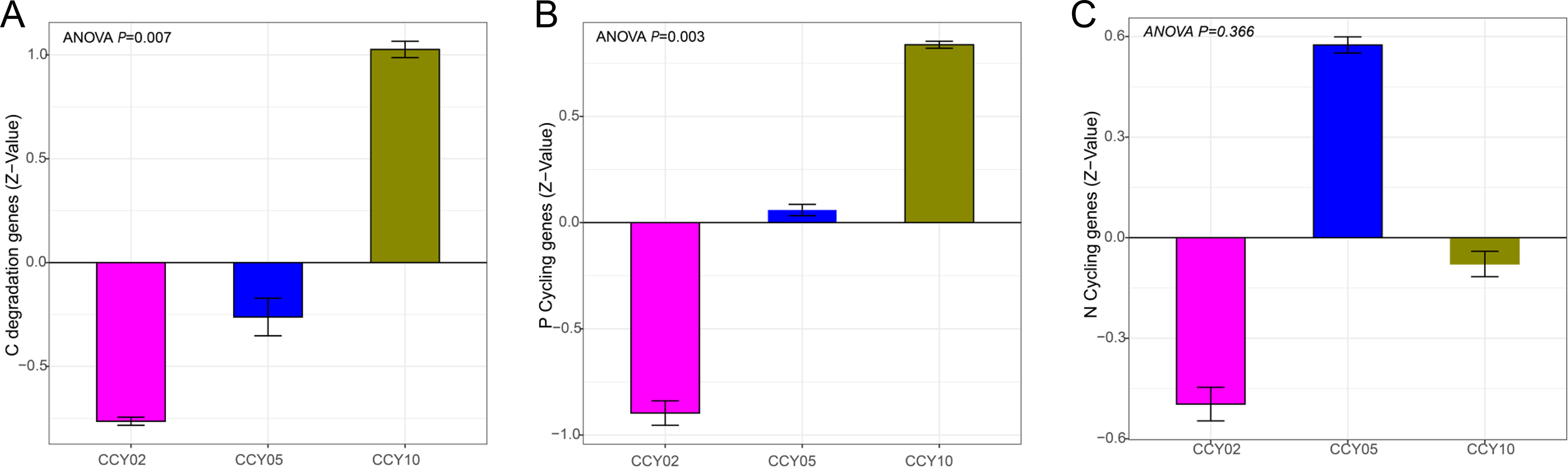

Supplement: Figure S5 — All of the genes were normalized in Z-value. P <0.05 was considered significant. [file peerj-10-13254-s005.png]

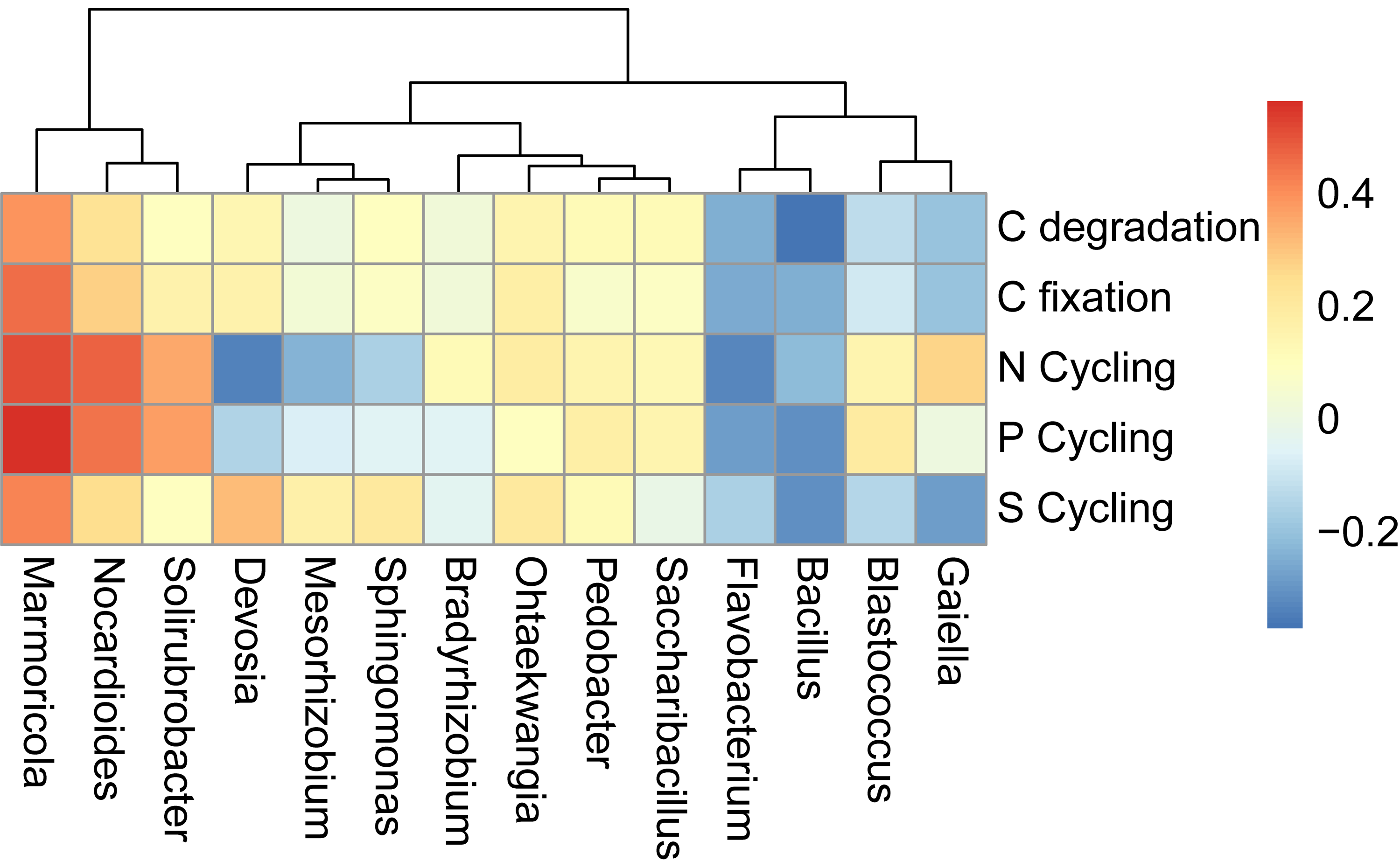

Supplement: Figure S6 — Values above/below zero represent positive/negative correlations. *P <0.05 was considered significant. [file peerj-10-13254-s006.png]

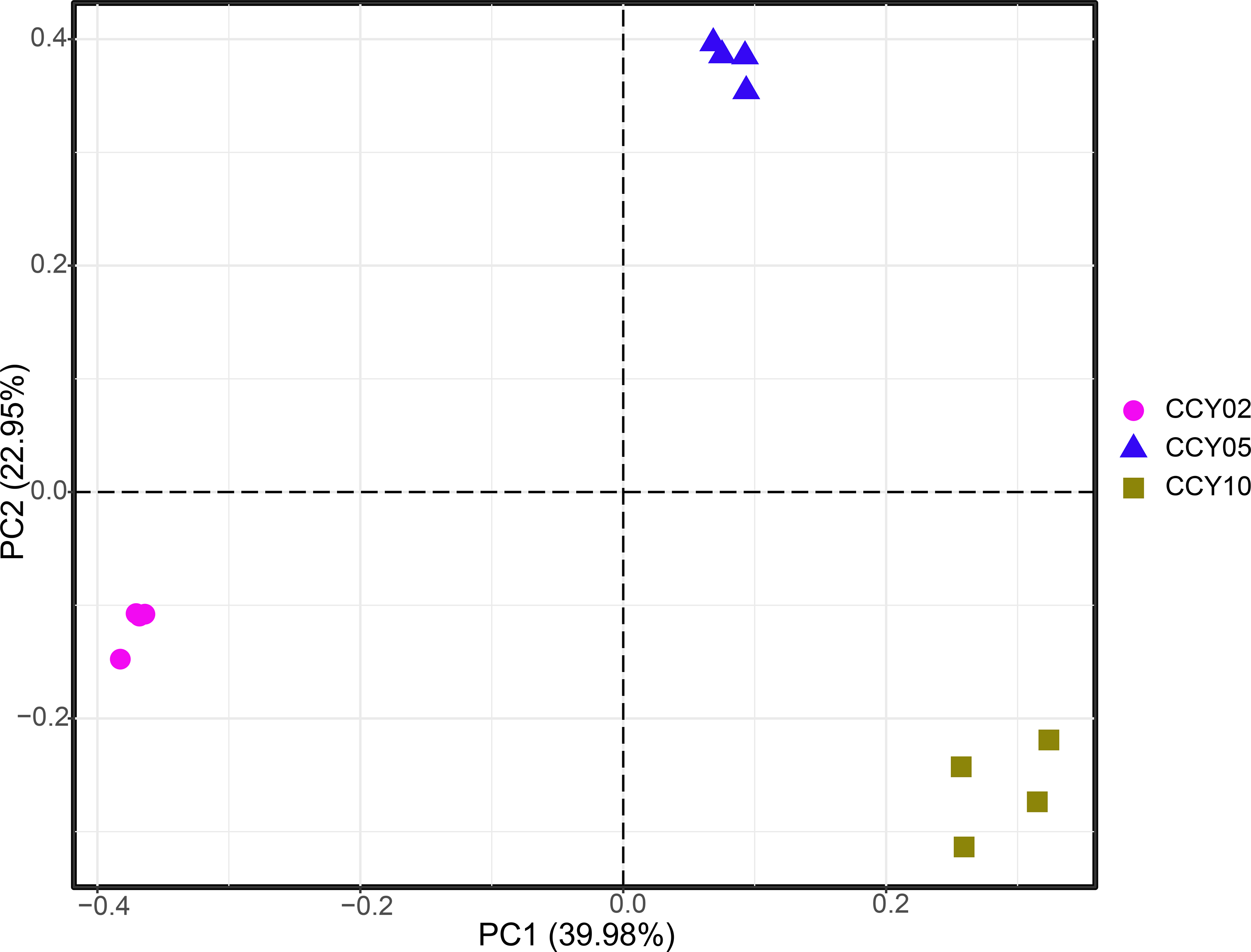

Supplement: Figure S7 [file peerj-10-13254-s007.png]

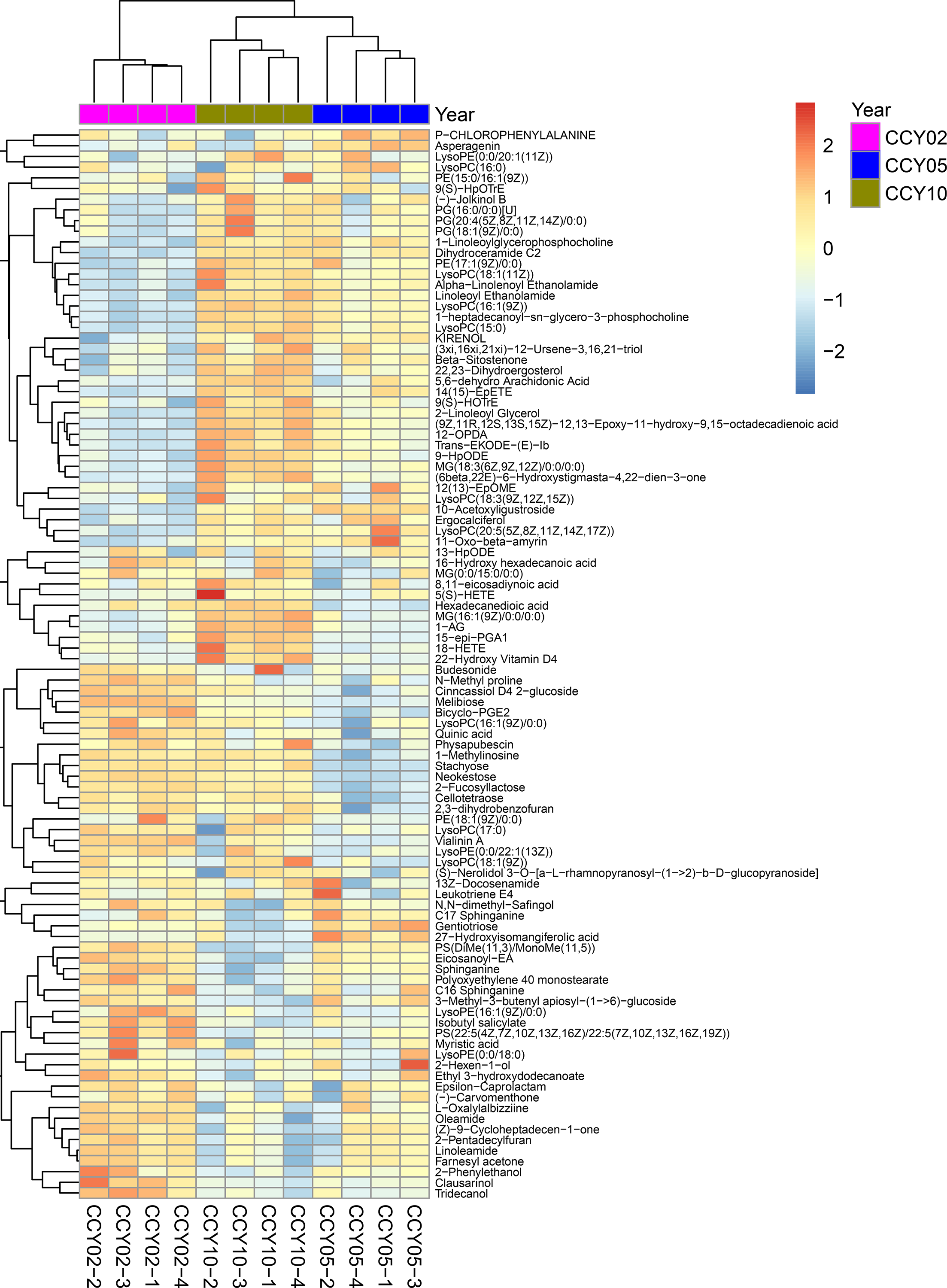

Supplement: Figure S8 [file peerj-10-13254-s008.png]
