# Supplementary material for: Soil metabolomics and bacterial functional traits revealed the responses of rhizosphere soil bacterial community to long-term continuous cropping of Tibetan barley
Source: PeerJ. 2022 Apr 7;10:e13254. doi: 10.7717/peerj.13254 (PMC8995024; doi:10.7717/peerj.13254)
Supplement: Table S1 [file peerj-10-13254-s009.docx]

**Table S1.** Significant analysis of communities’ dissimilarities between each group by Adonis analysis (*P* ＜0.05 considered significant).

| Group1 | Group2 | Adonis |
| --- | --- | --- |
| CCY02 | CCY10 | 0.044 |
| CCY02 | CCY05 | 0.031 |
| CCY10 | CCY05 | 0.029 |

The dissimilarities were calculated based on the ASV table between each paired groups.
