# Supplementary material for: Soil metabolomics and bacterial functional traits revealed the responses of rhizosphere soil bacterial community to long-term continuous cropping of Tibetan barley
Source: PeerJ. 2022 Apr 7;10:e13254. doi: 10.7717/peerj.13254 (PMC8995024; doi:10.7717/peerj.13254)
Supplement: Table S2 [file peerj-10-13254-s010.docx]

**Table S2.** Contribution of the communities’ similarities in pairwise comparison within the 14 overlapped main contributors by SIMPER analysis.

| Top contributors | CCY02 VS CCY05 | CCY02 VS CCY10 | CCY05 VS CCY10 |
| --- | --- | --- | --- |
| *Bacillus* | 3.93% | 3.97% | 1.80% |
| *Blastococcus* | 5.72% | 5.83% | 3.50% |
| *Bradyrhizobium* | 1.47% | 1.16% | 1.62% |
| *Devosia* | 1.12% | 2.12% | 1.77% |
| *Flavobacterium* | 2.22% | 2.05% | 2.27% |
| *Gaiella* | 2.62% | 1.28% | 3.94% |
| *Marmoricola* | 1.26% | 1.51% | 1.55% |
| *Mesorhizobium* | 1.10% | 1.19% | 0.94% |
| *Nocardioides* | 8.66% | 8.04% | 9.01% |
| *Ohtaekwangia* | 3.35% | 1.93% | 2.17% |
| *Pedobacter* | 3.02% | 1.66% | 2.14% |
| *Saccharibacillus* | 1.52% | 1.49% | 1.26% |
| *Solirubrobacter* | 1.95% | 1.94% | 1.52% |
| *Sphingomonas* | 3.59% | 4.72% | 3.41% |
