# Supplementary material for: Soil metabolomics and bacterial functional traits revealed the responses of rhizosphere soil bacterial community to long-term continuous cropping of Tibetan barley
Source: PeerJ. 2022 Apr 7;10:e13254. doi: 10.7717/peerj.13254 (PMC8995024; doi:10.7717/peerj.13254)
Supplement: Table S3 [file peerj-10-13254-s011.docx]

**Table S3.** The relative abundance of 164 core genera among samples.

| Core genera | CCY02_1 | CCY02_2 | CCY02_3 | CCY02_4 | CCY05_1 | CCY05_2 | CCY05_3 | CCY05_4 | CCY10_1 | CCY10_2 | CCY10_3 | CCY10_4 | Shared |
| --- | --- | --- | --- | --- | --- | --- | --- | --- | --- | --- | --- | --- | --- |
| *Sphingomonas* | 2.65% | 1.80% | 1.83% | 2.22% | 2.28% | 2.19% | 2.33% | 3.22% | 2.36% | 3.17% | 2.86% | 2.34% | Dominant_shared |
| *Solirubrobacter* | 0.99% | 1.12% | 1.46% | 0.72% | 1.06% | 1.29% | 0.77% | 0.88% | 0.84% | 1.19% | 0.92% | 1.00% | Dominant_shared |
| *Nocardioides* | 1.15% | 2.72% | 3.17% | 1.61% | 3.45% | 4.26% | 2.09% | 1.93% | 1.89% | 4.02% | 1.47% | 2.77% | Dominant_shared |
| *Blastococcus* | 2.76% | 2.98% | 4.02% | 1.87% | 2.66% | 3.01% | 1.74% | 2.16% | 2.24% | 2.61% | 2.55% | 2.22% | Dominant_shared |
| *Bacillus* | 1.35% | 1.50% | 1.90% | 0.96% | 0.67% | 1.04% | 0.81% | 0.96% | 0.82% | 1.43% | 0.82% | 0.83% | Dominant_shared |
| *Virgisporangium* | 0.06% | 0.04% | 0.06% | 0.03% | 0.05% | 0.03% | 0.03% | 0.02% | 0.03% | 0.02% | 0.03% | 0.05% | Rare_shared |
| *Vibrio* | 0.02% | 0.02% | 0.01% | 0.00% | 0.00% | 0.01% | 0.00% | 0.03% | 0.01% | 0.01% | 0.01% | 0.01% | Rare_shared |
| *Variovorax* | 0.35% | 0.19% | 0.31% | 0.29% | 0.22% | 0.26% | 0.29% | 0.29% | 0.29% | 0.25% | 0.23% | 0.25% | Rare_shared |
| *Thiobacter* | 0.03% | 0.01% | 0.03% | 0.03% | 0.03% | 0.01% | 0.03% | 0.04% | 0.02% | 0.03% | 0.02% | 0.00% | Rare_shared |
| *Thermomonas* | 0.07% | 0.02% | 0.03% | 0.09% | 0.01% | 0.00% | 0.00% | 0.01% | 0.05% | 0.06% | 0.04% | 0.04% | Rare_shared |
| *Thermoactinomyces* | 0.01% | 0.01% | 0.00% | 0.02% | 0.02% | 0.02% | 0.00% | 0.02% | 0.02% | 0.03% | 0.03% | 0.00% | Rare_shared |
| *Terrimonas* | 0.03% | 0.04% | 0.02% | 0.03% | 0.02% | 0.03% | 0.01% | 0.04% | 0.06% | 0.04% | 0.04% | 0.04% | Rare_shared |
| *Terrimicrobium* | 0.08% | 0.18% | 0.18% | 0.17% | 0.06% | 0.08% | 0.16% | 0.06% | 0.07% | 0.01% | 0.01% | 0.04% | Rare_shared |
| *Taibaiella* | 0.07% | 0.05% | 0.02% | 0.01% | 0.01% | 0.02% | 0.01% | 0.04% | 0.06% | 0.03% | 0.05% | 0.04% | Rare_shared |
| *Streptosporangium* | 0.08% | 0.20% | 0.21% | 0.18% | 0.14% | 0.13% | 0.14% | 0.19% | 0.14% | 0.23% | 0.07% | 0.11% | Rare_shared |
| *Streptomyces* | 0.75% | 0.68% | 0.79% | 0.51% | 0.70% | 0.78% | 0.75% | 1.08% | 0.68% | 0.77% | 0.83% | 0.62% | Rare_shared |
| *Steroidobacter* | 0.09% | 0.10% | 0.13% | 0.08% | 0.05% | 0.07% | 0.07% | 0.12% | 0.11% | 0.13% | 0.09% | 0.10% | Rare_shared |
| *Stenotrophomonas* | 0.02% | 0.01% | 0.02% | 0.00% | 0.01% | 0.00% | 0.01% | 0.00% | 0.02% | 0.01% | 0.03% | 0.02% | Rare_shared |
| *Stella* | 0.03% | 0.03% | 0.03% | 0.01% | 0.01% | 0.02% | 0.02% | 0.02% | 0.01% | 0.02% | 0.00% | 0.02% | Rare_shared |
| *Sporosarcina* | 0.03% | 0.04% | 0.06% | 0.03% | 0.02% | 0.02% | 0.02% | 0.02% | 0.01% | 0.03% | 0.02% | 0.01% | Rare_shared |
| *Sporichthya* | 0.20% | 0.21% | 0.29% | 0.12% | 0.15% | 0.15% | 0.11% | 0.13% | 0.20% | 0.22% | 0.22% | 0.14% | Rare_shared |
| *Sphingopyxis* | 0.22% | 0.10% | 0.06% | 0.22% | 0.18% | 0.14% | 0.16% | 0.35% | 0.24% | 0.28% | 0.29% | 0.20% | Rare_shared |
| *Sphingobacterium* | 0.01% | 0.01% | 0.03% | 0.01% | 0.00% | 0.01% | 0.03% | 0.04% | 0.03% | 0.02% | 0.02% | 0.02% | Rare_shared |
| *Sphaerobacter* | 0.03% | 0.01% | 0.03% | 0.03% | 0.03% | 0.02% | 0.01% | 0.01% | 0.00% | 0.01% | 0.00% | 0.02% | Rare_shared |
| *Skermanella* | 0.57% | 0.56% | 0.75% | 0.48% | 0.44% | 0.51% | 0.54% | 0.66% | 0.63% | 0.78% | 0.78% | 0.55% | Rare_shared |
| *Singulisphaera* | 0.09% | 0.29% | 0.21% | 0.11% | 0.13% | 0.17% | 0.09% | 0.05% | 0.11% | 0.07% | 0.09% | 0.17% | Rare_shared |
| *Segetibacter* | 0.03% | 0.01% | 0.02% | 0.03% | 0.02% | 0.05% | 0.05% | 0.02% | 0.01% | 0.04% | 0.02% | 0.00% | Rare_shared |
| *Sandaracinus* | 0.04% | 0.03% | 0.06% | 0.02% | 0.04% | 0.07% | 0.04% | 0.04% | 0.02% | 0.06% | 0.07% | 0.05% | Rare_shared |
| *Saccharibacillus* | 0.33% | 0.80% | 0.35% | 0.43% | 0.58% | 0.30% | 0.46% | 0.15% | 0.30% | 0.19% | 0.29% | 0.40% | Rare_shared |
| *Rufibacter* | 0.03% | 0.01% | 0.02% | 0.04% | 0.02% | 0.01% | 0.05% | 0.03% | 0.02% | 0.02% | 0.04% | 0.02% | Rare_shared |
| *Rubrobacter* | 0.36% | 0.33% | 0.26% | 0.29% | 0.24% | 0.26% | 0.31% | 0.19% | 0.44% | 0.26% | 0.53% | 0.50% | Rare_shared |
| *Rubellimicrobium* | 0.22% | 0.14% | 0.21% | 0.14% | 0.23% | 0.19% | 0.19% | 0.28% | 0.34% | 0.42% | 0.35% | 0.26% | Rare_shared |
| *Roseomonas* | 0.12% | 0.06% | 0.11% | 0.10% | 0.09% | 0.09% | 0.04% | 0.12% | 0.10% | 0.13% | 0.09% | 0.08% | Rare_shared |
| *Roseimicrobium* | 0.01% | 0.02% | 0.01% | 0.03% | 0.01% | 0.00% | 0.02% | 0.00% | 0.02% | 0.03% | 0.01% | 0.02% | Rare_shared |
| *Romboutsia* | 0.03% | 0.01% | 0.01% | 0.01% | 0.00% | 0.01% | 0.00% | 0.06% | 0.01% | 0.04% | 0.02% | 0.01% | Rare_shared |
| *Rhodovulum* | 0.01% | 0.01% | 0.01% | 0.02% | 0.02% | 0.02% | 0.01% | 0.02% | 0.01% | 0.03% | 0.04% | 0.02% | Rare_shared |
| *Rhodoplanes* | 0.29% | 0.22% | 0.27% | 0.28% | 0.20% | 0.18% | 0.27% | 0.37% | 0.28% | 0.34% | 0.28% | 0.24% | Rare_shared |
| *Rhodocytophaga* | 0.07% | 0.03% | 0.07% | 0.06% | 0.04% | 0.04% | 0.07% | 0.02% | 0.09% | 0.04% | 0.10% | 0.05% | Rare_shared |
| *Rhodococcus* | 0.30% | 0.21% | 0.33% | 0.22% | 0.15% | 0.15% | 0.22% | 0.32% | 0.17% | 0.16% | 0.16% | 0.17% | Rare_shared |
| *Rhizocola* | 0.01% | 0.01% | 0.03% | 0.00% | 0.01% | 0.02% | 0.02% | 0.00% | 0.02% | 0.01% | 0.00% | 0.01% | Rare_shared |
| *Rhizobium* | 0.36% | 0.24% | 0.37% | 0.27% | 0.33% | 0.27% | 0.35% | 0.48% | 0.35% | 0.41% | 0.44% | 0.26% | Rare_shared |
| *Rhizobacter* | 0.05% | 0.05% | 0.08% | 0.11% | 0.13% | 0.05% | 0.19% | 0.11% | 0.14% | 0.14% | 0.08% | 0.09% | Rare_shared |
| *Ramlibacter* | 0.53% | 0.25% | 0.26% | 0.43% | 0.29% | 0.35% | 0.35% | 0.55% | 0.30% | 0.41% | 0.37% | 0.36% | Rare_shared |
| *Pseudonocardia* | 0.38% | 0.45% | 0.61% | 0.33% | 0.25% | 0.32% | 0.22% | 0.25% | 0.25% | 0.39% | 0.29% | 0.25% | Rare_shared |
| *Pseudomonas* | 0.41% | 0.11% | 0.13% | 0.27% | 0.14% | 0.09% | 0.22% | 0.20% | 0.26% | 0.34% | 0.31% | 0.22% | Rare_shared |
| *Pseudolabrys* | 0.09% | 0.08% | 0.06% | 0.06% | 0.06% | 0.04% | 0.09% | 0.06% | 0.07% | 0.09% | 0.06% | 0.07% | Rare_shared |
| *Promicromonospora* | 0.03% | 0.04% | 0.10% | 0.04% | 0.20% | 0.09% | 0.13% | 0.06% | 0.08% | 0.04% | 0.08% | 0.07% | Rare_shared |
| *Pontibacter* | 0.27% | 0.16% | 0.16% | 0.16% | 0.16% | 0.19% | 0.20% | 0.35% | 0.18% | 0.22% | 0.23% | 0.22% | Rare_shared |
| *Polaromonas* | 0.11% | 0.04% | 0.04% | 0.07% | 0.04% | 0.03% | 0.03% | 0.07% | 0.10% | 0.06% | 0.06% | 0.12% | Rare_shared |
| *Planomicrobium* | 0.28% | 0.17% | 0.18% | 0.26% | 0.14% | 0.19% | 0.13% | 0.25% | 0.11% | 0.10% | 0.14% | 0.12% | Rare_shared |
| *Phyllobacterium* | 0.16% | 0.11% | 0.15% | 0.12% | 0.16% | 0.12% | 0.16% | 0.28% | 0.17% | 0.13% | 0.18% | 0.10% | Rare_shared |
| *Phenylobacterium* | 0.10% | 0.04% | 0.05% | 0.06% | 0.10% | 0.06% | 0.02% | 0.07% | 0.04% | 0.10% | 0.05% | 0.07% | Rare_shared |
| *Pedomicrobium* | 0.34% | 0.26% | 0.30% | 0.37% | 0.18% | 0.25% | 0.22% | 0.33% | 0.28% | 0.32% | 0.32% | 0.21% | Rare_shared |
| *Pedobacter* | 0.89% | 0.50% | 0.49% | 0.67% | 1.04% | 0.76% | 0.98% | 1.46% | 0.76% | 0.92% | 0.88% | 0.70% | Rare_shared |
| *Parviterribacter* | 0.14% | 0.16% | 0.24% | 0.07% | 0.23% | 0.19% | 0.13% | 0.17% | 0.14% | 0.24% | 0.20% | 0.18% | Rare_shared |
| *Paenibacillus* | 0.15% | 0.16% | 0.14% | 0.11% | 0.08% | 0.11% | 0.11% | 0.16% | 0.09% | 0.16% | 0.10% | 0.12% | Rare_shared |
| *Opitutus* | 0.04% | 0.00% | 0.01% | 0.04% | 0.02% | 0.02% | 0.02% | 0.05% | 0.05% | 0.04% | 0.01% | 0.03% | Rare_shared |
| *Ohtaekwangia* | 0.28% | 0.41% | 0.45% | 0.56% | 0.77% | 0.81% | 1.02% | 1.02% | 0.71% | 0.83% | 0.36% | 0.65% | Rare_shared |
| *Noviherbaspirillum* | 0.07% | 0.05% | 0.05% | 0.04% | 0.09% | 0.05% | 0.07% | 0.10% | 0.08% | 0.11% | 0.08% | 0.11% | Rare_shared |
| *Nostoc* | 0.05% | 0.14% | 0.16% | 0.08% | 0.07% | 0.08% | 0.13% | 0.11% | 0.09% | 0.10% | 0.13% | 0.07% | Rare_shared |
| *Nonomuraea* | 0.05% | 0.05% | 0.07% | 0.04% | 0.09% | 0.08% | 0.04% | 0.07% | 0.07% | 0.07% | 0.03% | 0.06% | Rare_shared |
| *Nocardia* | 0.06% | 0.02% | 0.02% | 0.06% | 0.09% | 0.04% | 0.06% | 0.01% | 0.05% | 0.03% | 0.04% | 0.01% | Rare_shared |
| *Nitrospira* | 0.53% | 0.41% | 0.49% | 0.48% | 0.47% | 0.48% | 0.59% | 0.89% | 0.56% | 0.57% | 0.51% | 0.43% | Rare_shared |
| *Nitrosospira* | 0.45% | 0.32% | 0.31% | 0.40% | 0.42% | 0.29% | 0.33% | 0.48% | 0.21% | 0.13% | 0.15% | 0.13% | Rare_shared |
| *Niastella* | 0.25% | 0.15% | 0.12% | 0.16% | 0.16% | 0.12% | 0.22% | 0.25% | 0.24% | 0.21% | 0.25% | 0.20% | Rare_shared |
| *Nakamurella* | 0.05% | 0.04% | 0.07% | 0.04% | 0.12% | 0.11% | 0.11% | 0.11% | 0.04% | 0.06% | 0.04% | 0.03% | Rare_shared |
| *Myxococcus* | 0.03% | 0.01% | 0.00% | 0.01% | 0.02% | 0.02% | 0.00% | 0.02% | 0.03% | 0.01% | 0.03% | 0.03% | Rare_shared |
| *Mycolicibacterium* | 0.33% | 0.20% | 0.33% | 0.21% | 0.30% | 0.17% | 0.19% | 0.28% | 0.26% | 0.36% | 0.25% | 0.25% | Rare_shared |
| *Mycobacterium* | 0.29% | 0.23% | 0.28% | 0.35% | 0.17% | 0.18% | 0.17% | 0.25% | 0.24% | 0.27% | 0.21% | 0.13% | Rare_shared |
| *Modestobacter* | 0.04% | 0.02% | 0.04% | 0.02% | 0.03% | 0.04% | 0.04% | 0.02% | 0.03% | 0.02% | 0.02% | 0.01% | Rare_shared |
| *Micromonospora* | 0.06% | 0.09% | 0.09% | 0.04% | 0.06% | 0.04% | 0.02% | 0.08% | 0.02% | 0.08% | 0.02% | 0.06% | Rare_shared |
| *Methylobacterium* | 0.03% | 0.06% | 0.09% | 0.04% | 0.03% | 0.05% | 0.06% | 0.04% | 0.05% | 0.04% | 0.03% | 0.05% | Rare_shared |
| *Mesorhizobium* | 0.57% | 0.38% | 0.41% | 0.35% | 0.52% | 0.53% | 0.53% | 0.69% | 0.63% | 0.70% | 0.42% | 0.45% | Rare_shared |
| *Massilia* | 0.05% | 0.02% | 0.02% | 0.08% | 0.08% | 0.06% | 0.07% | 0.10% | 0.05% | 0.06% | 0.05% | 0.07% | Rare_shared |
| *Marmoricola* | 0.31% | 0.58% | 0.68% | 0.40% | 0.63% | 0.78% | 0.53% | 0.41% | 0.42% | 0.90% | 0.41% | 0.48% | Rare_shared |
| *Lysobacter* | 0.39% | 0.18% | 0.12% | 0.26% | 0.30% | 0.25% | 0.29% | 0.26% | 0.33% | 0.39% | 0.26% | 0.33% | Rare_shared |
| *Lysinibacillus* | 0.06% | 0.10% | 0.08% | 0.03% | 0.03% | 0.05% | 0.07% | 0.05% | 0.01% | 0.07% | 0.01% | 0.04% | Rare_shared |
| *Luteolibacter* | 0.06% | 0.04% | 0.06% | 0.16% | 0.04% | 0.05% | 0.08% | 0.02% | 0.12% | 0.00% | 0.04% | 0.12% | Rare_shared |
| *Longimicrobium* | 0.10% | 0.07% | 0.10% | 0.07% | 0.11% | 0.06% | 0.04% | 0.06% | 0.14% | 0.06% | 0.15% | 0.09% | Rare_shared |
| *Litorilinea* | 0.01% | 0.01% | 0.00% | 0.01% | 0.02% | 0.01% | 0.01% | 0.02% | 0.01% | 0.01% | 0.05% | 0.02% | Rare_shared |
| *Levilinea* | 0.19% | 0.38% | 0.49% | 0.21% | 0.24% | 0.40% | 0.36% | 0.11% | 0.28% | 0.11% | 0.26% | 0.34% | Rare_shared |
| *Lentzea* | 0.12% | 0.16% | 0.20% | 0.09% | 0.17% | 0.12% | 0.16% | 0.19% | 0.12% | 0.09% | 0.14% | 0.13% | Rare_shared |
| *Legionella* | 0.03% | 0.00% | 0.01% | 0.04% | 0.03% | 0.01% | 0.01% | 0.01% | 0.06% | 0.08% | 0.05% | 0.04% | Rare_shared |
| *Lacibacter* | 0.05% | 0.02% | 0.02% | 0.03% | 0.01% | 0.04% | 0.01% | 0.02% | 0.04% | 0.03% | 0.03% | 0.01% | Rare_shared |
| *Kribbella* | 0.13% | 0.10% | 0.24% | 0.12% | 0.21% | 0.27% | 0.29% | 0.32% | 0.09% | 0.14% | 0.14% | 0.12% | Rare_shared |
| *Kouleothrix* | 0.09% | 0.03% | 0.05% | 0.10% | 0.05% | 0.05% | 0.04% | 0.12% | 0.10% | 0.13% | 0.11% | 0.08% | Rare_shared |
| *Kineosporia* | 0.04% | 0.08% | 0.06% | 0.06% | 0.06% | 0.07% | 0.06% | 0.01% | 0.03% | 0.02% | 0.06% | 0.08% | Rare_shared |
| *Ilumatobacter* | 0.70% | 0.70% | 0.78% | 0.61% | 0.77% | 0.79% | 0.52% | 0.70% | 0.69% | 0.82% | 0.78% | 0.70% | Rare_shared |
| *Iamia* | 0.10% | 0.14% | 0.19% | 0.16% | 0.17% | 0.18% | 0.09% | 0.18% | 0.12% | 0.22% | 0.18% | 0.19% | Rare_shared |
| *Hyphomicrobium* | 0.18% | 0.14% | 0.15% | 0.10% | 0.05% | 0.10% | 0.15% | 0.18% | 0.12% | 0.18% | 0.11% | 0.16% | Rare_shared |
| *Hymenobacter* | 0.07% | 0.02% | 0.04% | 0.02% | 0.05% | 0.04% | 0.07% | 0.07% | 0.07% | 0.09% | 0.05% | 0.08% | Rare_shared |
| *Hydrogenophaga* | 0.02% | 0.01% | 0.01% | 0.03% | 0.00% | 0.02% | 0.01% | 0.01% | 0.05% | 0.07% | 0.05% | 0.03% | Rare_shared |
| *Herpetosiphon* | 0.02% | 0.03% | 0.02% | 0.06% | 0.02% | 0.03% | 0.05% | 0.04% | 0.09% | 0.10% | 0.04% | 0.09% | Rare_shared |
| *Haliangium* | 0.16% | 0.07% | 0.08% | 0.09% | 0.10% | 0.12% | 0.07% | 0.15% | 0.17% | 0.13% | 0.20% | 0.14% | Rare_shared |
| *Glycomyces* | 0.02% | 0.01% | 0.03% | 0.01% | 0.12% | 0.09% | 0.07% | 0.02% | 0.01% | 0.01% | 0.00% | 0.03% | Rare_shared |
| *Geodermatophilus* | 0.01% | 0.03% | 0.08% | 0.02% | 0.03% | 0.01% | 0.02% | 0.02% | 0.03% | 0.04% | 0.04% | 0.03% | Rare_shared |
| *Geobacter* | 0.01% | 0.01% | 0.01% | 0.01% | 0.01% | 0.03% | 0.02% | 0.01% | 0.01% | 0.02% | 0.01% | 0.00% | Rare_shared |
| *Gemmatimonas* | 0.29% | 0.17% | 0.14% | 0.32% | 0.25% | 0.29% | 0.30% | 0.57% | 0.36% | 0.36% | 0.36% | 0.33% | Rare_shared |
| *Gemmata* | 0.07% | 0.10% | 0.08% | 0.09% | 0.06% | 0.05% | 0.04% | 0.00% | 0.10% | 0.03% | 0.09% | 0.06% | Rare_shared |
| *Gaiella* | 1.07% | 0.79% | 0.86% | 0.79% | 1.15% | 1.24% | 1.19% | 1.43% | 0.63% | 0.91% | 0.79% | 0.65% | Rare_shared |
| *Flavobacterium* | 1.05% | 0.42% | 0.59% | 0.95% | 0.50% | 0.40% | 0.38% | 0.65% | 0.82% | 0.66% | 1.03% | 0.51% | Rare_shared |
| *Flavitalea* | 0.13% | 0.07% | 0.03% | 0.09% | 0.08% | 0.12% | 0.10% | 0.19% | 0.12% | 0.13% | 0.13% | 0.10% | Rare_shared |
| *Flavisolibacter* | 0.40% | 0.18% | 0.12% | 0.31% | 0.27% | 0.27% | 0.41% | 0.57% | 0.33% | 0.43% | 0.34% | 0.29% | Rare_shared |
| *Fictibacillus* | 0.00% | 0.01% | 0.01% | 0.01% | 0.01% | 0.01% | 0.01% | 0.01% | 0.02% | 0.04% | 0.01% | 0.01% | Rare_shared |
| *Ferruginibacter* | 0.23% | 0.08% | 0.05% | 0.20% | 0.07% | 0.03% | 0.08% | 0.18% | 0.15% | 0.20% | 0.16% | 0.15% | Rare_shared |
| *Ferrimicrobium* | 0.04% | 0.04% | 0.05% | 0.07% | 0.04% | 0.02% | 0.01% | 0.04% | 0.06% | 0.06% | 0.04% | 0.02% | Rare_shared |
| *Effusibacillus* | 0.04% | 0.06% | 0.01% | 0.05% | 0.03% | 0.02% | 0.04% | 0.05% | 0.01% | 0.02% | 0.00% | 0.01% | Rare_shared |
| *Edaphobaculum* | 0.02% | 0.01% | 0.01% | 0.01% | 0.00% | 0.01% | 0.03% | 0.00% | 0.01% | 0.02% | 0.03% | 0.03% | Rare_shared |
| *Dyadobacter* | 0.05% | 0.07% | 0.06% | 0.11% | 0.05% | 0.06% | 0.04% | 0.04% | 0.10% | 0.05% | 0.06% | 0.05% | Rare_shared |
| *Dongia* | 0.10% | 0.03% | 0.06% | 0.07% | 0.06% | 0.04% | 0.07% | 0.06% | 0.07% | 0.07% | 0.09% | 0.03% | Rare_shared |
| *Dokdonella* | 0.08% | 0.05% | 0.02% | 0.05% | 0.02% | 0.02% | 0.01% | 0.01% | 0.05% | 0.07% | 0.08% | 0.08% | Rare_shared |
| *Devosia* | 0.78% | 0.58% | 0.57% | 0.72% | 0.57% | 0.63% | 0.80% | 1.01% | 0.95% | 1.05% | 0.94% | 0.84% | Rare_shared |
| *Desulfovirga* | 0.24% | 0.22% | 0.19% | 0.27% | 0.26% | 0.21% | 0.28% | 0.19% | 0.25% | 0.16% | 0.16% | 0.18% | Rare_shared |
| *Desulfoglaeba* | 0.04% | 0.03% | 0.04% | 0.05% | 0.06% | 0.06% | 0.03% | 0.07% | 0.03% | 0.06% | 0.02% | 0.04% | Rare_shared |
| *Desulfocaldus* | 0.02% | 0.03% | 0.03% | 0.03% | 0.04% | 0.07% | 0.04% | 0.02% | 0.03% | 0.03% | 0.02% | 0.06% | Rare_shared |
| *Derxia* | 0.14% | 0.13% | 0.15% | 0.11% | 0.13% | 0.17% | 0.09% | 0.15% | 0.13% | 0.14% | 0.11% | 0.07% | Rare_shared |
| *Dehalogenimonas* | 0.02% | 0.01% | 0.03% | 0.02% | 0.01% | 0.01% | 0.01% | 0.00% | 0.00% | 0.01% | 0.01% | 0.01% | Rare_shared |
| *Dactylosporangium* | 0.08% | 0.08% | 0.04% | 0.05% | 0.00% | 0.04% | 0.01% | 0.02% | 0.06% | 0.09% | 0.06% | 0.13% | Rare_shared |
| *Craurococcus* | 0.02% | 0.03% | 0.03% | 0.01% | 0.03% | 0.05% | 0.03% | 0.00% | 0.03% | 0.01% | 0.05% | 0.02% | Rare_shared |
| *Conexibacter* | 0.08% | 0.15% | 0.23% | 0.08% | 0.09% | 0.16% | 0.11% | 0.10% | 0.10% | 0.14% | 0.13% | 0.09% | Rare_shared |
| *Cohnella* | 0.01% | 0.07% | 0.03% | 0.03% | 0.03% | 0.10% | 0.05% | 0.03% | 0.03% | 0.03% | 0.04% | 0.00% | Rare_shared |
| *Clostridium* | 0.02% | 0.06% | 0.10% | 0.05% | 0.01% | 0.02% | 0.01% | 0.02% | 0.04% | 0.06% | 0.02% | 0.06% | Rare_shared |
| *Chryseobacterium* | 0.08% | 0.02% | 0.02% | 0.03% | 0.06% | 0.02% | 0.04% | 0.07% | 0.00% | 0.01% | 0.04% | 0.00% | Rare_shared |
| *Chondromyces* | 0.02% | 0.01% | 0.01% | 0.01% | 0.01% | 0.01% | 0.05% | 0.01% | 0.01% | 0.02% | 0.03% | 0.02% | Rare_shared |
| *Chloroflexus* | 0.29% | 0.25% | 0.31% | 0.24% | 0.24% | 0.22% | 0.24% | 0.18% | 0.17% | 0.27% | 0.22% | 0.22% | Rare_shared |
| *Chitinophaga* | 0.02% | 0.04% | 0.01% | 0.03% | 0.02% | 0.01% | 0.04% | 0.05% | 0.02% | 0.04% | 0.01% | 0.03% | Rare_shared |
| *Chelatococcus* | 0.06% | 0.01% | 0.02% | 0.06% | 0.05% | 0.07% | 0.04% | 0.03% | 0.04% | 0.08% | 0.04% | 0.05% | Rare_shared |
| *Cellvibrio* | 0.14% | 0.05% | 0.09% | 0.07% | 0.04% | 0.07% | 0.08% | 0.12% | 0.17% | 0.10% | 0.11% | 0.13% | Rare_shared |
| *Cellulomonas* | 0.08% | 0.18% | 0.39% | 0.19% | 0.17% | 0.10% | 0.11% | 0.05% | 0.27% | 0.12% | 0.32% | 0.29% | Rare_shared |
| *Catenulispora* | 0.16% | 0.22% | 0.18% | 0.17% | 0.17% | 0.22% | 0.18% | 0.10% | 0.21% | 0.11% | 0.28% | 0.23% | Rare_shared |
| *Catellatospora* | 0.02% | 0.01% | 0.02% | 0.01% | 0.00% | 0.01% | 0.02% | 0.02% | 0.00% | 0.01% | 0.01% | 0.01% | Rare_shared |
| *Candidatus Solibacter* | 0.07% | 0.04% | 0.04% | 0.06% | 0.04% | 0.03% | 0.02% | 0.06% | 0.06% | 0.07% | 0.05% | 0.03% | Rare_shared |
| *Candidatus Phytoplasma* | 0.31% | 0.24% | 0.23% | 0.24% | 0.13% | 0.18% | 0.13% | 0.37% | 0.25% | 0.32% | 0.24% | 0.25% | Rare_shared |
| *Candidatus Halyseosphaera* | 0.41% | 0.34% | 0.41% | 0.27% | 0.30% | 0.27% | 0.20% | 0.25% | 0.18% | 0.30% | 0.25% | 0.30% | Rare_shared |
| *Candidatus Entotheonella* | 0.03% | 0.02% | 0.03% | 0.07% | 0.04% | 0.04% | 0.04% | 0.07% | 0.04% | 0.05% | 0.03% | 0.02% | Rare_shared |
| *Caenimonas* | 0.01% | 0.01% | 0.01% | 0.02% | 0.01% | 0.01% | 0.04% | 0.04% | 0.03% | 0.01% | 0.02% | 0.01% | Rare_shared |
| *Brevundimonas* | 0.14% | 0.09% | 0.09% | 0.16% | 0.08% | 0.06% | 0.09% | 0.12% | 0.12% | 0.15% | 0.17% | 0.12% | Rare_shared |
| *Brevibacillus* | 0.06% | 0.08% | 0.04% | 0.07% | 0.04% | 0.09% | 0.06% | 0.10% | 0.03% | 0.05% | 0.03% | 0.01% | Rare_shared |
| *Bradyrhizobium* | 0.92% | 0.57% | 0.74% | 0.70% | 0.66% | 0.57% | 0.84% | 1.16% | 0.62% | 0.84% | 0.92% | 0.63% | Rare_shared |
| *Bosea* | 0.06% | 0.05% | 0.10% | 0.13% | 0.12% | 0.07% | 0.07% | 0.17% | 0.13% | 0.13% | 0.08% | 0.08% | Rare_shared |
| *Blastocatella* | 0.16% | 0.30% | 0.14% | 0.27% | 0.09% | 0.11% | 0.20% | 0.01% | 0.15% | 0.07% | 0.12% | 0.15% | Rare_shared |
| *Bdellovibrio* | 0.07% | 0.05% | 0.04% | 0.04% | 0.03% | 0.04% | 0.05% | 0.07% | 0.04% | 0.04% | 0.04% | 0.05% | Rare_shared |
| *Bauldia* | 0.28% | 0.23% | 0.26% | 0.18% | 0.21% | 0.24% | 0.24% | 0.26% | 0.14% | 0.31% | 0.23% | 0.21% | Rare_shared |
| *Bacteriovorax* | 0.09% | 0.06% | 0.07% | 0.03% | 0.03% | 0.03% | 0.03% | 0.04% | 0.05% | 0.07% | 0.03% | 0.05% | Rare_shared |
| *Arthrobacter* | 0.05% | 0.08% | 0.14% | 0.07% | 0.06% | 0.04% | 0.02% | 0.01% | 0.06% | 0.08% | 0.05% | 0.05% | Rare_shared |
| *Arenimonas* | 0.41% | 0.26% | 0.16% | 0.35% | 0.35% | 0.30% | 0.29% | 0.44% | 0.35% | 0.38% | 0.36% | 0.35% | Rare_shared |
| *Arenimicrobium* | 0.12% | 0.32% | 0.16% | 0.23% | 0.31% | 0.22% | 0.29% | 0.08% | 0.19% | 0.11% | 0.24% | 0.20% | Rare_shared |
| *Archangium* | 0.01% | 0.01% | 0.02% | 0.01% | 0.01% | 0.01% | 0.01% | 0.04% | 0.01% | 0.02% | 0.03% | 0.01% | Rare_shared |
| *Aquimonas* | 0.14% | 0.11% | 0.05% | 0.12% | 0.11% | 0.14% | 0.17% | 0.19% | 0.18% | 0.22% | 0.23% | 0.17% | Rare_shared |
| *Aquihabitans* | 0.04% | 0.03% | 0.06% | 0.05% | 0.01% | 0.01% | 0.02% | 0.02% | 0.04% | 0.04% | 0.05% | 0.04% | Rare_shared |
| *Aquicella* | 0.04% | 0.01% | 0.02% | 0.03% | 0.03% | 0.02% | 0.03% | 0.02% | 0.04% | 0.03% | 0.02% | 0.04% | Rare_shared |
| *Amaricoccus* | 0.11% | 0.10% | 0.23% | 0.05% | 0.12% | 0.07% | 0.06% | 0.10% | 0.10% | 0.15% | 0.14% | 0.10% | Rare_shared |
| *Altererythrobacter* | 0.07% | 0.05% | 0.03% | 0.08% | 0.04% | 0.04% | 0.05% | 0.13% | 0.09% | 0.09% | 0.10% | 0.06% | Rare_shared |
| *Allocatelliglobosispora* | 0.02% | 0.02% | 0.02% | 0.02% | 0.01% | 0.02% | 0.01% | 0.03% | 0.02% | 0.04% | 0.01% | 0.01% | Rare_shared |
| *Aliihoeflea* | 0.04% | 0.04% | 0.05% | 0.06% | 0.09% | 0.10% | 0.07% | 0.12% | 0.04% | 0.10% | 0.05% | 0.04% | Rare_shared |
| *Agromyces* | 0.26% | 0.27% | 0.33% | 0.19% | 0.29% | 0.29% | 0.18% | 0.23% | 0.29% | 0.34% | 0.29% | 0.19% | Rare_shared |
| *Aeromicrobium* | 0.24% | 0.30% | 0.59% | 0.28% | 0.66% | 0.51% | 0.47% | 0.26% | 0.26% | 0.30% | 0.35% | 0.29% | Rare_shared |
| *Adhaeribacter* | 0.26% | 0.17% | 0.13% | 0.33% | 0.23% | 0.18% | 0.22% | 0.41% | 0.26% | 0.24% | 0.14% | 0.17% | Rare_shared |
| *Actinoplanes* | 0.26% | 0.28% | 0.35% | 0.16% | 0.14% | 0.18% | 0.09% | 0.24% | 0.20% | 0.23% | 0.16% | 0.23% | Rare_shared |
| *Actinomadura* | 0.05% | 0.02% | 0.05% | 0.03% | 0.01% | 0.00% | 0.02% | 0.01% | 0.00% | 0.03% | 0.01% | 0.00% | Rare_shared |
| *Actinocorallia* | 0.02% | 0.03% | 0.04% | 0.03% | 0.03% | 0.05% | 0.04% | 0.05% | 0.03% | 0.02% | 0.03% | 0.01% | Rare_shared |
| *Acidovorax* | 0.18% | 0.06% | 0.10% | 0.22% | 0.27% | 0.10% | 0.26% | 0.44% | 0.17% | 0.15% | 0.16% | 0.16% | Rare_shared |
| *Acidobacterium* | 0.05% | 0.04% | 0.02% | 0.05% | 0.04% | 0.02% | 0.05% | 0.03% | 0.04% | 0.02% | 0.02% | 0.03% | Rare_shared |
| *Aciditerrimonas* | 0.15% | 0.09% | 0.17% | 0.13% | 0.14% | 0.13% | 0.14% | 0.20% | 0.11% | 0.12% | 0.09% | 0.08% | Rare_shared |

Taxa (at the genus level) present at all samples at more or ＜1% mean relative abundance was defined as dominant or rare core taxa, respectively.
