# Supplementary material for: Soil metabolomics and bacterial functional traits revealed the responses of rhizosphere soil bacterial community to long-term continuous cropping of Tibetan barley
Source: PeerJ. 2022 Apr 7;10:e13254. doi: 10.7717/peerj.13254 (PMC8995024; doi:10.7717/peerj.13254)
Supplement: Table S4 [file peerj-10-13254-s012.docx]

**Table S4.** Relative abundance of each gene among samples by QMEC analysis.

| Cateories | Gene_name | CCY02_1 | CCY02_2 | CCY02_3 | CCY02_4 | CCY05_1 | CCY05_2 | CCY05_3 | CCY05_4 | CCY10_1 | CCY10_2 | CCY10_3 | CCY10_4 |
| --- | --- | --- | --- | --- | --- | --- | --- | --- | --- | --- | --- | --- | --- |
| C degradation | abfA | 259,826.42 | 261,984.30 | 212,927.42 | 239,612.35 | 344,895.78 | 298,559.06 | 233,062.82 | 221,007.66 | 258,521.26 | 307,376.80 | 367,441.55 | 305,018.56 |
| C degradation | xylA | 196,528.68 | 219,647.74 | 207,894.42 | 208,372.01 | 255,150.94 | 279,557.43 | 300,488.93 | 30,764.35 | 259,072.08 | 297,957.91 | 302,050.99 | 324,556.96 |
| C degradation | manA | 112,005.18 | 122,558.33 | 127,123.24 | 115,931.07 | 137,194.94 | 131,346.52 | 137,266.86 | 140,813.44 | 195,697.44 | 255,568.36 | 234,481.67 | 213,564.48 |
| C degradation | gmGDH | 126,422.35 | 125,145.94 | 130,886.21 | 136,477.31 | 155,257.50 | 137,415.67 | 167,009.85 | 169,462.79 | 133,276.03 | 153,568.63 | 142,242.19 | 137,682.63 |
| C degradation | gam | 110,307.67 | 117,950.82 | 129,889.71 | 107,754.20 | 140,559.90 | 96,653.18 | 125,124.19 | 128,223.61 | 148,732.12 | 198,512.42 | 169,130.90 | 166,740.75 |
| C degradation | mnp | 42,651.49 | 52,288.91 | 51,094.79 | 45,274.79 | 79,282.38 | 80,123.35 | 97,855.46 | 8,452.74 | 90,012.47 | 102,132.16 | 95,204.53 | 87,492.08 |
| C degradation | exg | 37,972.40 | 33,923.09 | 40,903.42 | 34,395.83 | 29,173.28 | 30,584.69 | 27,590.90 | 25,357.87 | 26,798.00 | 29,958.79 | 36,646.06 | 34,940.54 |
| C degradation | glx | 12,693.79 | 14,167.74 | 14,124.66 | 12,595.03 | 26,859.50 | 24,280.73 | 28,773.12 | 21,056.30 | 31,814.36 | 44,925.36 | 33,930.52 | 35,272.19 |
| C degradation | IsoP | 7,966.71 | 8,497.70 | 80,337.67 | 7,846.07 | 13,100.61 | 9,973.43 | 14,004.10 | 10,955.39 | 15,869.28 | 17,919.43 | 18,228.97 | 19,070.11 |
| C degradation | chiA | 9,059.05 | 9,825.22 | 10,478.21 | 9,376.53 | 5,838.37 | 7,176.82 | 68,421.01 | 6,243.84 | 10,905.60 | 17,009.07 | 17,728.88 | 15,345.20 |
| C degradation | lig | 11,311.09 | 11,009.23 | 10,482.83 | 9,946.63 | 10,817.36 | 9,890.17 | 13,745.76 | 11,374.50 | 21,546.93 | 24,695.22 | 24,062.00 | 25,742.85 |
| C degradation | CDH | 6,121.67 | 6,705.11 | 6,900.60 | 5,776.89 | 9,654.98 | 12,844.17 | 10,814.00 | 11,448.08 | 12,496.31 | 13,692.93 | 14,654.75 | 12,841.35 |
| C degradation | amyA | 1,461.34 | 1,324.25 | 1,262.77 | 1,181.46 | 1,725.60 | 1,597.54 | 2,064.13 | 1,997.55 | 3,736.82 | 4,568.01 | 4,264.69 | 3,913.53 |
| C degradation | apu | 1,643.64 | 1,778.50 | 1,850.70 | 1,590.98 | 1,973.38 | 1,810.18 | 1,292.14 | 1,539.71 | 2,498.64 | 3,568.27 | 3,037.26 | 2,870.97 |
| C degradation | naglu | 114.27 | 455.48 | 104.28 | 149.75 | 120.60 | 126.55 | 360.69 | 0.00 | 236.77 | 679.52 | 283.72 | 312.04 |
| C degradation | pox | 196.37 | 239.07 | 231.74 | 0.00 | 107.00 | 214.91 | 462.53 | 0.00 | 200.96 | 363.93 | 463.89 | 450.44 |
| C degradation | amyX | 0.00 | 0.00 | 0.00 | 0.00 | 74.39 | 0.00 | 75.24 | 0.00 | 123.69 | 134.96 | 125.26 | 170.54 |
| C fixation | acsA | 1,735,463.17 | 1,705,892.13 | 2,108,715.88 | 1,615,883.64 | 1,959,660.25 | 1,699,576.00 | 1,661,696.93 | 1,785,038.03 | 1,588,907.89 | 2,313,263.72 | 2,331,328.84 | 1,963,178.50 |
| C fixation | mct | 574,585.69 | 593,043.43 | 600,359.47 | 579,069.75 | 987,941.52 | 814,987.80 | 898,150.96 | 91,307.21 | 886,316.78 | 976,246.52 | 1,011,147.11 | 946,920.58 |
| C fixation | rbcL | 306,644.26 | 297,807.37 | 333,009.12 | 342,346.93 | 401,498.88 | 368,292.90 | 336,935.86 | 389,799.17 | 221,484.12 | 293,273.33 | 332,649.47 | 262,261.44 |
| C fixation | acsE | 193,088.55 | 224,988.33 | 207,726.08 | 198,493.82 | 268,783.51 | 203,254.27 | 441,351.39 | 44,114.09 | 346,193.29 | 390,214.05 | 401,349.77 | 366,166.75 |
| C fixation | korA | 86,975.69 | 91,698.03 | 884,023.59 | 90,375.40 | 121,206.39 | 118,922.64 | 177,540.46 | 25,142.14 | 176,054.94 | 159,528.31 | 168,827.17 | 158,516.42 |
| C fixation | frdA | 112,862.67 | 114,064.86 | 131,693.78 | 117,179.81 | 134,832.77 | 124,166.14 | 133,348.42 | 119,330.60 | 99,943.16 | 115,807.82 | 110,860.04 | 109,872.72 |
| C fixation | smtA | 74,958.95 | 79,301.02 | 77,565.21 | 76,267.95 | 100,682.23 | 95,152.35 | 115,971.29 | 111,041.20 | 147,086.94 | 150,466.71 | 148,243.25 | 158,155.05 |
| C fixation | pccA | 73,110.74 | 71,411.55 | 70,871.37 | 62,646.21 | 84,763.88 | 79,369.85 | 74,533.90 | 78,176.37 | 78,117.82 | 80,201.27 | 72,304.02 | 76,546.29 |
| C fixation | accA | 26,009.63 | 30,313.31 | 28,548.97 | 29,073.13 | 29,981.53 | 30,071.37 | 27,162.79 | 23,596.31 | 25,074.15 | 29,159.21 | 30,989.66 | 25,981.34 |
| C fixation | aclB | 20,129.77 | 20,034.38 | 21,989.71 | 19,574.25 | 23,804.84 | 17,094.13 | 23,830.31 | 22,200.14 | 39,656.95 | 44,535.88 | 41,929.42 | 39,423.71 |
| C fixation | acsB | 388.81 | 545.61 | 368.72 | 210.72 | 471.36 | 355.47 | 540.61 | 305.16 | 817.58 | 946.47 | 1,037.64 | 970.19 |
| C fixation | mcrA | 0.00 | 0.00 | 0.00 | 0.00 | 0.00 | 58.56 | 0.00 | 0.00 | 197.09 | 56.00 | 0.00 | 0.00 |
| C fixation | cdaR | 0.00 | 0.00 | 0.00 | 0.00 | 0.00 | 0.00 | 0.00 | 0.00 | 68.86 | 86.29 | 52.51 | 0.00 |
| N Cycling | UreC | 2,014,648.68 | 1,930,723.83 | 2,676,033.68 | 2,251,114.69 | 2,731,688.06 | 2,365,288.66 | 2,165,308.12 | 2,481,117.59 | 1,991,441.32 | 2,269,013.91 | 2,555,509.66 | 2,169,970.53 |
| N Cycling | amoA2 | 284,988.73 | 292,707.16 | 318,459.37 | 294,901.18 | 208,407.74 | 286,512.57 | 293,000.19 | 277,818.68 | 102,457.28 | 115,099.31 | 127,667.16 | 105,538.04 |
| N Cycling | gdh | 106,754.92 | 119,533.47 | 117,229.53 | 111,442.29 | 134,518.62 | 142,637.83 | 158,822.88 | 137,348.39 | 175,037.71 | 179,400.85 | 163,178.89 | 166,452.68 |
| N Cycling | amoA1 | 45,390.03 | 45,802.71 | 49,290.51 | 47,916.48 | 46,418.52 | 58,005.14 | 68,007.47 | 52,600.22 | 81,672.71 | 85,566.54 | 70,905.76 | 83,785.06 |
| N Cycling | nifH | 48,737.83 | 51,361.15 | 49,152.10 | 50,611.15 | 61,402.38 | 59,439.69 | 65,999.41 | 52,234.65 | 68,633.52 | 70,386.62 | 73,433.67 | 72,556.09 |
| N Cycling | napA | 37,923.35 | 40,286.28 | 39,470.23 | 38,377.25 | 57,985.88 | 46,818.79 | 65,930.68 | 58,727.14 | 55,043.23 | 61,334.80 | 60,308.87 | 59,341.17 |
| N Cycling | nxrA | 52,175.91 | 56,257.27 | 59,535.65 | 57,381.23 | 44,198.25 | 50,784.98 | 48,690.33 | 45,953.65 | 37,018.00 | 39,552.31 | 42,114.48 | 34,151.48 |
| N Cycling | nosZ1 | 29,445.97 | 31,767.87 | 32,961.20 | 29,844.49 | 44,251.08 | 39,089.90 | 41,369.34 | 46,280.42 | 65,658.62 | 70,916.99 | 68,190.93 | 59,354.28 |
| N Cycling | nirS1 | 24,840.64 | 25,012.71 | 28,773.22 | 24,367.53 | 34,527.30 | 34,441.11 | 36,582.30 | 32,491.83 | 50,363.77 | 57,275.89 | 55,395.79 | 49,115.85 |
| N Cycling | nirK3 | 27,796.12 | 26,141.28 | 25,813.53 | 24,309.99 | 36,891.25 | 39,475.69 | 34,184.66 | 31,719.21 | 40,569.17 | 54,475.69 | 59,244.28 | 50,462.90 |
| N Cycling | amoB | 21,020.98 | 24,595.45 | 23,059.51 | 23,011.46 | 28,478.29 | 24,038.01 | 25,275.21 | 31,651.95 | 42,872.69 | 60,382.86 | 57,521.24 | 53,182.95 |
| N Cycling | nirS2 | 9,768.65 | 10,247.11 | 10,840.42 | 9,179.47 | 16,567.21 | 12,625.72 | 10,442.96 | 13,261.06 | 19,593.23 | 22,169.67 | 25,137.50 | 21,288.68 |
| N Cycling | nirK1 | 5,170.03 | 5,793.56 | 5,477.54 | 4,972.25 | 9,131.51 | 7,812.96 | 8,787.92 | 8,835.09 | 9,599.90 | 9,885.29 | 10,268.41 | 9,839.14 |
| N Cycling | nirS3 | 5,060.66 | 6,547.95 | 5,949.49 | 5,627.24 | 5,581.19 | 5,170.32 | 5,429.24 | 5,774.55 | 9,906.36 | 11,077.25 | 12,666.50 | 10,435.57 |
| N Cycling | narG | 4,995.61 | 5,445.03 | 5,735.59 | 3,745.83 | 6,699.70 | 6,581.59 | 6,927.94 | 7,098.16 | 8,540.34 | 8,224.02 | 10,773.83 | 9,269.78 |
| N Cycling | nirK2 | 1,877.21 | 1,741.01 | 1,950.59 | 1,862.96 | 3,316.00 | 3,763.94 | 4,008.50 | 3,232.67 | 4,174.21 | 5,063.56 | 6,138.42 | 4,368.82 |
| N Cycling | hzsB | 1,375.72 | 1,278.11 | 1,090.38 | 1,231.82 | 2,960.00 | 3,033.44 | 3,552.36 | 3,236.01 | 2,997.07 | 4,566.14 | 3,872.31 | 3,695.89 |
| N Cycling | nasA | 598.53 | 675.39 | 572.55 | 134.88 | 633.25 | 424.97 | 932.44 | 471.90 | 1,852.79 | 1,075.80 | 1,186.35 | 1,853.29 |
| N Cycling | hao | 350.54 | 496.48 | 472.30 | 294.57 | 666.85 | 627.01 | 814.57 | 758.96 | 785.03 | 1,090.63 | 836.58 | 1,064.45 |
| P Cycling | bpp | 7,235.13 | 8,514.08 | 9,329.57 | 7,292.50 | 8,498.72 | 7,307.67 | 7,928.49 | 8,963.58 | 9,143.69 | 11,287.58 | 11,092.12 | 11,214.48 |
| P Cycling | cphy | 0.00 | 45.62 | 0.00 | 0.00 | 52.31 | 52.90 | 126.13 | 0.00 | 150.40 | 205.43 | 178.74 | 151.70 |
| P Cycling | phnK | 361,563.14 | 367,400.47 | 466,363.85 | 322,650.13 | 384,422.41 | 403,000.21 | 480,102.90 | 398,609.89 | 456,092.74 | 509,734.26 | 497,872.05 | 453,132.54 |
| P Cycling | phoD | 446,614.48 | 467,476.91 | 594,421.22 | 448,340.87 | 514,723.09 | 537,206.97 | 563,830.32 | 545,471.65 | 565,126.90 | 590,167.77 | 533,427.78 | 557,457.90 |
| P Cycling | phoX | 13,755.35 | 14,775.57 | 14,926.23 | 13,134.48 | 23,484.13 | 25,492.17 | 20,363.63 | 2,237.53 | 27,865.40 | 29,431.75 | 31,644.35 | 26,189.19 |
| P Cycling | pqqC | 40,019.40 | 42,374.59 | 38,584.03 | 44,473.23 | 54,686.10 | 49,169.23 | 59,306.90 | 53,361.77 | 53,967.03 | 57,490.16 | 52,113.04 | 54,810.68 |
| S Cycling | apsA | 82,155.37 | 913,449.54 | 99,855.86 | 90,850.16 | 93,039.64 | 83,109.30 | 95,798.63 | 97,784.60 | 174,339.02 | 168,652.63 | 182,112.02 | 172,784.59 |
| S Cycling | dsrA | 5,795.01 | 5,996.39 | 6,533.36 | 5,297.09 | 5,751.68 | 5,745.19 | 5,236.22 | 5,257.08 | 9,406.62 | 10,750.73 | 8,834.84 | 9,539.20 |
| S Cycling | dsrB | 204.58 | 220.72 | 178.48 | 283.12 | 290.38 | 296.80 | 226.68 | 291.93 | 580.41 | 593.87 | 593.89 | 713.98 |
| S Cycling | SoxY | 86,148.07 | 89,574.06 | 79,787.57 | 73,743.38 | 105,320.84 | 101,264.53 | 117,705.26 | 11,985.59 | 177,857.69 | 169,358.76 | 190,276.38 | 170,988.31 |
| S Cycling | YedZ | 84,955.59 | 104,461.43 | 104,155.65 | 91,911.63 | 159,928.35 | 154,149.10 | 154,133.07 | 15,620.05 | 141,466.28 | 173,901.73 | 189,212.25 | 167,386.85 |
