# Supplementary material for: Soil metabolomics and bacterial functional traits revealed the responses of rhizosphere soil bacterial community to long-term continuous cropping of Tibetan barley
Source: PeerJ. 2022 Apr 7;10:e13254. doi: 10.7717/peerj.13254 (PMC8995024; doi:10.7717/peerj.13254)
Supplement: Table S5 [file peerj-10-13254-s013.docx]

**Table S5.** Compare the abundance of each cycling pathway among different continuous cropping years by Z-value analysis.

|  | Items | F value | *P* value |
| --- | --- | --- | --- |
| Z-value (unitless) | C-degradation | 9.25 | 0.007* |
|  | C-fixation | 1.896 | 0.206 |
|  | P-cycling | 5.351 | 0.029* |
|  | N-cycling | 1.128 | 0.366 |
|  | S-cycling | 0.89 | 0.444 |

*P* ＜0.05 considered significant.
