# Supplementary material for: Soil metabolomics and bacterial functional traits revealed the responses of rhizosphere soil bacterial community to long-term continuous cropping of Tibetan barley
Source: PeerJ. 2022 Apr 7;10:e13254. doi: 10.7717/peerj.13254 (PMC8995024; doi:10.7717/peerj.13254)
Supplement: Table S6 [file peerj-10-13254-s014.docx]

**Table S6.** Correlation between the Shannon index and each main functional category by Spearman’s correlation test.

| Functional categories | r | *P* |
| --- | --- | --- |
| C degradation | -0.01 | 0.98 |
| C fixation | -0.05 | 0.87 |
| N Cycling | -0.71 | 0.01* |
| P Cycling | -0.30 | 0.34 |
| S Cycling | -0.23 | 0.46 |
| CNPS | -0.20 | 0.53 |

*P* ＜0.05 considered significant.
