# Supplementary material for: Soil metabolomics and bacterial functional traits revealed the responses of rhizosphere soil bacterial community to long-term continuous cropping of Tibetan barley
Source: PeerJ. 2022 Apr 7;10:e13254. doi: 10.7717/peerj.13254 (PMC8995024; doi:10.7717/peerj.13254)
Supplement: Table S7 [file peerj-10-13254-s015.docx]

**Table S7.** Specific interaction type between each main genus and the functional genes.

| source | target | interactionType | *p*-Val | weight |
| --- | --- | --- | --- | --- |
| aclB | 1Saccharothrix | copresence | 0.00 | 0.85 |
| acsB | 1Saccharothrix | copresence | 0.00 | 0.92 |
| acsE | 1Saccharothrix | copresence | 0.00 | 0.81 |
| apsA | 1Saccharothrix | copresence | 0.00 | 0.83 |
| CDH | 1Saccharothrix | copresence | 0.01 | 0.66 |
| chiA | 1Saccharothrix | copresence | 0.00 | 0.83 |
| gdh | 1Saccharothrix | copresence | 0.00 | 0.72 |
| glx | 1Saccharothrix | copresence | 0.00 | 0.70 |
| IsoP | 1Saccharothrix | copresence | 0.00 | 0.81 |
| korA | 1Saccharothrix | copresence | 0.00 | 0.77 |
| lig | 1Saccharothrix | copresence | 0.00 | 0.85 |
| manA | 1Saccharothrix | copresence | 0.00 | 0.85 |
| napA | 1Saccharothrix | copresence | 0.00 | 0.73 |
| nifH | 1Saccharothrix | copresence | 0.00 | 0.83 |
| nosZ1 | 1Saccharothrix | copresence | 0.00 | 0.80 |
| SoxY | 1Saccharothrix | copresence | 0.00 | 0.79 |
| xylA | 1Saccharothrix | copresence | 0.00 | 0.79 |
| 1Saccharothrix | amoA1 | copresence | 0.00 | 0.78 |
| 1Saccharothrix | amoB | copresence | 0.00 | 0.83 |
| 1Saccharothrix | amyA | copresence | 0.00 | 0.84 |
| 1Saccharothrix | amyX | copresence | 0.00 | 0.88 |
| 1Saccharothrix | apu | copresence | 0.00 | 0.77 |
| 1Saccharothrix | bpp | copresence | 0.00 | 0.71 |
| 1Saccharothrix | cphy | copresence | 0.00 | 0.74 |
| 1Saccharothrix | dsrA | copresence | 0.00 | 0.79 |
| 1Saccharothrix | dsrB | copresence | 0.00 | 0.95 |
| 1Saccharothrix | gam | copresence | 0.00 | 0.82 |
| 1Saccharothrix | hao | copresence | 0.00 | 0.78 |
| 1Saccharothrix | hzsB | copresence | 0.00 | 0.74 |
| 1Saccharothrix | mct | copresence | 0.00 | 0.79 |
| 1Saccharothrix | mnp | copresence | 0.00 | 0.83 |
| 1Saccharothrix | naglu | copresence | 0.00 | 0.73 |
| 1Saccharothrix | narG | copresence | 0.00 | 0.76 |
| 1Saccharothrix | nasA | copresence | 0.00 | 0.85 |
| 1Saccharothrix | nirK1 | copresence | 0.00 | 0.83 |
| 1Saccharothrix | nirK2 | copresence | 0.00 | 0.81 |
| 1Saccharothrix | nirS1 | copresence | 0.00 | 0.79 |
| 1Saccharothrix | nirS2 | copresence | 0.00 | 0.81 |
| 1Saccharothrix | nirS3 | copresence | 0.00 | 0.84 |
| 1Saccharothrix | phoX | copresence | 0.00 | 0.85 |
| 1Saccharothrix | pox | copresence | 0.00 | 0.70 |
| 1Saccharothrix | pqqC | copresence | 0.01 | 0.66 |
| 1Saccharothrix | smtA | copresence | 0.00 | 0.86 |
| 1Saccharothrix | YedZ | copresence | 0.00 | 0.79 |
| aclB | 2Planomicrobium | mutualExclusion | 0.00 | -0.78 |
| acsB | 2Planomicrobium | mutualExclusion | 0.00 | -0.77 |
| acsE | 2Planomicrobium | mutualExclusion | 0.00 | -0.83 |
| amoA1 | 2Planomicrobium | mutualExclusion | 0.00 | -0.83 |
| amyX | 2Planomicrobium | mutualExclusion | 0.00 | -0.86 |
| apsA | 2Planomicrobium | mutualExclusion | 0.00 | -0.86 |
| bpp | 2Planomicrobium | mutualExclusion | 0.00 | -0.80 |
| chiA | 2Planomicrobium | mutualExclusion | 0.00 | -0.80 |
| IsoP | 2Planomicrobium | mutualExclusion | 0.00 | -0.83 |
| mct | 2Planomicrobium | mutualExclusion | 0.00 | -0.86 |
| napA | 2Planomicrobium | mutualExclusion | 0.00 | -0.78 |
| narG | 2Planomicrobium | mutualExclusion | 0.00 | -0.85 |
| nasA | 2Planomicrobium | mutualExclusion | 0.00 | -0.80 |
| nosZ1 | 2Planomicrobium | mutualExclusion | 0.00 | -0.81 |
| phoX | 2Planomicrobium | mutualExclusion | 0.00 | -0.80 |
| SoxY | 2Planomicrobium | mutualExclusion | 0.00 | -0.84 |
| 2Planomicrobium | amoB | mutualExclusion | 0.00 | -0.87 |
| 2Planomicrobium | amyA | mutualExclusion | 0.00 | -0.82 |
| 2Planomicrobium | apu | mutualExclusion | 0.00 | -0.87 |
| 2Planomicrobium | CDH | mutualExclusion | 0.00 | -0.73 |
| 2Planomicrobium | cphy | mutualExclusion | 0.00 | -0.91 |
| 2Planomicrobium | dsrA | mutualExclusion | 0.00 | -0.76 |
| 2Planomicrobium | gam | mutualExclusion | 0.00 | -0.84 |
| 2Planomicrobium | gdh | mutualExclusion | 0.00 | -0.72 |
| 2Planomicrobium | glx | mutualExclusion | 0.00 | -0.74 |
| 2Planomicrobium | hao | mutualExclusion | 0.00 | -0.87 |
| 2Planomicrobium | hzsB | mutualExclusion | 0.00 | -0.83 |
| 2Planomicrobium | korA | mutualExclusion | 0.00 | -0.86 |
| 2Planomicrobium | lig | mutualExclusion | 0.00 | -0.83 |
| 2Planomicrobium | manA | mutualExclusion | 0.00 | -0.83 |
| 2Planomicrobium | mnp | mutualExclusion | 0.00 | -0.86 |
| 2Planomicrobium | naglu | mutualExclusion | 0.01 | -0.67 |
| 2Planomicrobium | nifH | mutualExclusion | 0.00 | -0.82 |
| 2Planomicrobium | nirK1 | mutualExclusion | 0.00 | -0.80 |
| 2Planomicrobium | nirK2 | mutualExclusion | 0.00 | -0.80 |
| 2Planomicrobium | nirK3 | mutualExclusion | 0.00 | -0.74 |
| 2Planomicrobium | nirS1 | mutualExclusion | 0.00 | -0.80 |
| 2Planomicrobium | nirS2 | mutualExclusion | 0.00 | -0.82 |
| 2Planomicrobium | nirS3 | mutualExclusion | 0.00 | -0.81 |
| 2Planomicrobium | smtA | mutualExclusion | 0.00 | -0.79 |
| 2Planomicrobium | xylA | mutualExclusion | 0.00 | -0.71 |
| 2Planomicrobium | YedZ | mutualExclusion | 0.00 | -0.87 |
| aclB | 3Edaphobaculum | copresence | 0.00 | 0.75 |
| acsA | 3Edaphobaculum | copresence | 0.01 | 0.65 |
| acsB | 3Edaphobaculum | copresence | 0.00 | 0.77 |
| acsE | 3Edaphobaculum | copresence | 0.00 | 0.73 |
| apsA | 3Edaphobaculum | copresence | 0.00 | 0.72 |
| bpp | 3Edaphobaculum | copresence | 0.00 | 0.72 |
| chiA | 3Edaphobaculum | copresence | 0.00 | 0.74 |
| IsoP | 3Edaphobaculum | copresence | 0.00 | 0.73 |
| lig | 3Edaphobaculum | copresence | 0.00 | 0.72 |
| mnp | 3Edaphobaculum | copresence | 0.00 | 0.72 |
| narG | 3Edaphobaculum | copresence | 0.00 | 0.72 |
| nasA | 3Edaphobaculum | copresence | 0.00 | 0.73 |
| nirS2 | 3Edaphobaculum | copresence | 0.00 | 0.74 |
| nirS3 | 3Edaphobaculum | copresence | 0.00 | 0.74 |
| smtA | 3Edaphobaculum | copresence | 0.00 | 0.76 |
| 3Edaphobaculum | amoA1 | copresence | 0.00 | 0.75 |
| 3Edaphobaculum | amoB | copresence | 0.00 | 0.72 |
| 3Edaphobaculum | amyA | copresence | 0.00 | 0.78 |
| 3Edaphobaculum | apu | copresence | 0.00 | 0.72 |
| 3Edaphobaculum | CDH | copresence | 0.00 | 0.76 |
| 3Edaphobaculum | dsrA | copresence | 0.00 | 0.72 |
| 3Edaphobaculum | dsrB | copresence | 0.00 | 0.79 |
| 3Edaphobaculum | gam | copresence | 0.00 | 0.73 |
| 3Edaphobaculum | glx | copresence | 0.00 | 0.78 |
| 3Edaphobaculum | hao | copresence | 0.00 | 0.74 |
| 3Edaphobaculum | hzsB | copresence | 0.00 | 0.77 |
| 3Edaphobaculum | korA | copresence | 0.00 | 0.73 |
| 3Edaphobaculum | manA | copresence | 0.00 | 0.73 |
| 3Edaphobaculum | mct | copresence | 0.00 | 0.72 |
| 3Edaphobaculum | napA | copresence | 0.00 | 0.72 |
| 3Edaphobaculum | nifH | copresence | 0.01 | 0.68 |
| 3Edaphobaculum | nirK1 | copresence | 0.00 | 0.77 |
| 3Edaphobaculum | nirK2 | copresence | 0.00 | 0.72 |
| 3Edaphobaculum | nirS1 | copresence | 0.00 | 0.70 |
| 3Edaphobaculum | nosZ1 | copresence | 0.00 | 0.75 |
| 3Edaphobaculum | phoX | copresence | 0.00 | 0.77 |
| 3Edaphobaculum | pox | copresence | 0.00 | 0.81 |
| 3Edaphobaculum | SoxY | copresence | 0.00 | 0.73 |
| 3Edaphobaculum | xylA | copresence | 0.00 | 0.75 |
| 3Edaphobaculum | YedZ | copresence | 0.00 | 0.73 |
| amoB | 4Nitrosospira | mutualExclusion | 0.00 | -0.76 |
| amyX | 4Nitrosospira | mutualExclusion | 0.00 | -0.72 |
| dsrA | 4Nitrosospira | mutualExclusion | 0.00 | -0.76 |
| korA | 4Nitrosospira | mutualExclusion | 0.00 | -0.74 |
| lig | 4Nitrosospira | mutualExclusion | 0.00 | -0.73 |
| mct | 4Nitrosospira | mutualExclusion | 0.01 | -0.67 |
| mnp | 4Nitrosospira | mutualExclusion | 0.00 | -0.77 |
| narG | 4Nitrosospira | mutualExclusion | 0.00 | -0.76 |
| nirS1 | 4Nitrosospira | mutualExclusion | 0.00 | -0.76 |
| nirS2 | 4Nitrosospira | mutualExclusion | 0.00 | -0.80 |
| nirS3 | 4Nitrosospira | mutualExclusion | 0.00 | -0.83 |
| xylA | 4Nitrosospira | mutualExclusion | 0.01 | -0.66 |
| 4Nitrosospira | aclB | mutualExclusion | 0.01 | -0.67 |
| 4Nitrosospira | acsB | mutualExclusion | 0.00 | -0.71 |
| 4Nitrosospira | acsE | mutualExclusion | 0.01 | -0.66 |
| 4Nitrosospira | amoA1 | mutualExclusion | 0.01 | -0.69 |
| 4Nitrosospira | amyA | mutualExclusion | 0.01 | -0.66 |
| 4Nitrosospira | apsA | mutualExclusion | 0.00 | -0.77 |
| 4Nitrosospira | apu | mutualExclusion | 0.00 | -0.73 |
| 4Nitrosospira | bpp | mutualExclusion | 0.00 | -0.71 |
| 4Nitrosospira | cdaR | mutualExclusion | 0.01 | -0.65 |
| 4Nitrosospira | CDH | mutualExclusion | 0.00 | -0.76 |
| 4Nitrosospira | cphy | mutualExclusion | 0.00 | -0.80 |
| 4Nitrosospira | gdh | mutualExclusion | 0.00 | -0.71 |
| 4Nitrosospira | glx | mutualExclusion | 0.00 | -0.72 |
| 4Nitrosospira | hao | mutualExclusion | 0.01 | -0.69 |
| 4Nitrosospira | hzsB | mutualExclusion | 0.00 | -0.78 |
| 4Nitrosospira | IsoP | mutualExclusion | 0.01 | -0.66 |
| 4Nitrosospira | manA | mutualExclusion | 0.00 | -0.74 |
| 4Nitrosospira | nifH | mutualExclusion | 0.01 | -0.69 |
| 4Nitrosospira | nirK1 | mutualExclusion | 0.00 | -0.71 |
| 4Nitrosospira | nirK2 | mutualExclusion | 0.00 | -0.71 |
| 4Nitrosospira | nirK3 | mutualExclusion | 0.00 | -0.81 |
| 4Nitrosospira | nosZ1 | mutualExclusion | 0.00 | -0.76 |
| 4Nitrosospira | phoX | mutualExclusion | 0.00 | -0.71 |
| 4Nitrosospira | pox | mutualExclusion | 0.00 | -0.70 |
| 4Nitrosospira | smtA | mutualExclusion | 0.00 | -0.70 |
| 4Nitrosospira | SoxY | mutualExclusion | 0.00 | -0.71 |
| 4Nitrosospira | YedZ | mutualExclusion | 0.00 | -0.73 |
| apu | 5Angustibacter | mutualExclusion | 0.01 | -0.67 |
| bpp | 5Angustibacter | mutualExclusion | 0.00 | -0.75 |
| chiA | 5Angustibacter | mutualExclusion | 0.00 | -0.78 |
| IsoP | 5Angustibacter | mutualExclusion | 0.00 | -0.85 |
| mct | 5Angustibacter | mutualExclusion | 0.00 | -0.75 |
| mnp | 5Angustibacter | mutualExclusion | 0.01 | -0.69 |
| napA | 5Angustibacter | mutualExclusion | 0.01 | -0.67 |
| nirK1 | 5Angustibacter | mutualExclusion | 0.00 | -0.75 |
| nirK2 | 5Angustibacter | mutualExclusion | 0.01 | -0.69 |
| nirS2 | 5Angustibacter | mutualExclusion | 0.01 | -0.69 |
| nirS3 | 5Angustibacter | mutualExclusion | 0.00 | -0.75 |
| xylA | 5Angustibacter | mutualExclusion | 0.00 | -0.83 |
| 5Angustibacter | abfA | mutualExclusion | 0.01 | -0.68 |
| 5Angustibacter | aclB | mutualExclusion | 0.01 | -0.68 |
| 5Angustibacter | acsB | mutualExclusion | 0.00 | -0.73 |
| 5Angustibacter | acsE | mutualExclusion | 0.00 | -0.80 |
| 5Angustibacter | amoA1 | mutualExclusion | 0.00 | -0.73 |
| 5Angustibacter | amoB | mutualExclusion | 0.01 | -0.67 |
| 5Angustibacter | amyA | mutualExclusion | 0.01 | -0.68 |
| 5Angustibacter | apsA | mutualExclusion | 0.01 | -0.69 |
| 5Angustibacter | dsrA | mutualExclusion | 0.00 | -0.73 |
| 5Angustibacter | gam | mutualExclusion | 0.00 | -0.73 |
| 5Angustibacter | gdh | mutualExclusion | 0.00 | -0.76 |
| 5Angustibacter | hao | mutualExclusion | 0.01 | -0.69 |
| 5Angustibacter | lig | mutualExclusion | 0.01 | -0.68 |
| 5Angustibacter | manA | mutualExclusion | 0.00 | -0.72 |
| 5Angustibacter | nasA | mutualExclusion | 0.00 | -0.79 |
| 5Angustibacter | nifH | mutualExclusion | 0.00 | -0.75 |
| 5Angustibacter | nirS1 | mutualExclusion | 0.01 | -0.69 |
| 5Angustibacter | nosZ1 | mutualExclusion | 0.01 | -0.69 |
| 5Angustibacter | phoD | mutualExclusion | 0.00 | -0.71 |
| 5Angustibacter | phoX | mutualExclusion | 0.00 | -0.77 |
| 5Angustibacter | pox | mutualExclusion | 0.00 | -0.74 |
| 5Angustibacter | smtA | mutualExclusion | 0.00 | -0.85 |
| 5Angustibacter | SoxY | mutualExclusion | 0.01 | -0.68 |
| aclB | 6Sanguibacter | copresence | 0.01 | 0.65 |
| acsB | 6Sanguibacter | copresence | 0.00 | 0.80 |
| amoB | 6Sanguibacter | copresence | 0.00 | 0.70 |
| amyX | 6Sanguibacter | copresence | 0.00 | 0.83 |
| cphy | 6Sanguibacter | copresence | 0.00 | 0.73 |
| lig | 6Sanguibacter | copresence | 0.00 | 0.74 |
| manA | 6Sanguibacter | copresence | 0.00 | 0.71 |
| mnp | 6Sanguibacter | copresence | 0.00 | 0.70 |
| nasA | 6Sanguibacter | copresence | 0.01 | 0.66 |
| nifH | 6Sanguibacter | copresence | 0.01 | 0.69 |
| nirK2 | 6Sanguibacter | copresence | 0.01 | 0.66 |
| nirK3 | 6Sanguibacter | copresence | 0.00 | 0.78 |
| nirS1 | 6Sanguibacter | copresence | 0.01 | 0.69 |
| nirS2 | 6Sanguibacter | copresence | 0.00 | 0.72 |
| nirS3 | 6Sanguibacter | copresence | 0.00 | 0.75 |
| nosZ1 | 6Sanguibacter | copresence | 0.01 | 0.65 |
| phoX | 6Sanguibacter | copresence | 0.01 | 0.67 |
| smtA | 6Sanguibacter | copresence | 0.01 | 0.69 |
| 6Sanguibacter | apsA | copresence | 0.00 | 0.70 |
| 6Sanguibacter | dsrA | copresence | 0.01 | 0.66 |
| aclB | 7Brevibacillus | mutualExclusion | 0.00 | -0.75 |
| acsB | 7Brevibacillus | mutualExclusion | 0.00 | -0.71 |
| chiA | 7Brevibacillus | mutualExclusion | 0.01 | -0.69 |
| nosZ1 | 7Brevibacillus | mutualExclusion | 0.01 | -0.65 |
| SoxY | 7Brevibacillus | mutualExclusion | 0.01 | -0.67 |
| xylA | 7Brevibacillus | mutualExclusion | 0.00 | -0.73 |
| 7Brevibacillus | acsE | mutualExclusion | 0.01 | -0.66 |
| 7Brevibacillus | amyX | mutualExclusion | 0.00 | -0.76 |
| 7Brevibacillus | dsrA | mutualExclusion | 0.00 | -0.75 |
| 7Brevibacillus | gam | mutualExclusion | 0.01 | -0.67 |
| 7Brevibacillus | IsoP | mutualExclusion | 0.00 | -0.71 |
| 7Brevibacillus | lig | mutualExclusion | 0.00 | -0.82 |
| 7Brevibacillus | nasA | mutualExclusion | 0.01 | -0.68 |
| 7Brevibacillus | nifH | mutualExclusion | 0.01 | -0.65 |
| 7Brevibacillus | nirK3 | mutualExclusion | 0.00 | -0.74 |
| 7Brevibacillus | nirS1 | mutualExclusion | 0.01 | -0.69 |
| 7Brevibacillus | nirS2 | mutualExclusion | 0.00 | -0.70 |
| 7Brevibacillus | nirS3 | mutualExclusion | 0.01 | -0.67 |
| 7Brevibacillus | smtA | mutualExclusion | 0.01 | -0.65 |
| amoB | 8Caulobacter | mutualExclusion | 0.01 | -0.65 |
| apu | 8Caulobacter | mutualExclusion | 0.01 | -0.65 |
| cphy | 8Caulobacter | mutualExclusion | 0.00 | -0.71 |
| hzsB | 8Caulobacter | mutualExclusion | 0.01 | -0.69 |
| korA | 8Caulobacter | mutualExclusion | 0.01 | -0.69 |
| nirK3 | 8Caulobacter | mutualExclusion | 0.01 | -0.65 |
| nirS2 | 8Caulobacter | mutualExclusion | 0.01 | -0.69 |
| YedZ | 8Caulobacter | mutualExclusion | 0.01 | -0.69 |
| 8Caulobacter | CDH | mutualExclusion | 0.01 | -0.65 |
| 8Caulobacter | hao | mutualExclusion | 0.01 | -0.69 |
| 8Caulobacter | manA | mutualExclusion | 0.01 | -0.65 |
| 8Caulobacter | napA | mutualExclusion | 0.01 | -0.69 |
| 8Caulobacter | nirK1 | mutualExclusion | 0.01 | -0.69 |
| 8Caulobacter | nirK2 | mutualExclusion | 0.01 | -0.69 |
| 8Caulobacter | nirS1 | mutualExclusion | 0.01 | -0.69 |
| 8Caulobacter | nosZ1 | mutualExclusion | 0.01 | -0.69 |
| nirK3 | 9Pantoea | copresence | 0.01 | 0.69 |
| nirS1 | 9Pantoea | copresence | 0.01 | 0.66 |
| nirS2 | 9Pantoea | copresence | 0.01 | 0.69 |
| nirS3 | 9Pantoea | copresence | 0.01 | 0.69 |
| rbcL | 9Pantoea | mutualExclusion | 0.01 | -0.66 |
| 9Pantoea | aclB | copresence | 0.00 | 0.70 |
| 9Pantoea | acsB | copresence | 0.00 | 0.76 |
| 9Pantoea | amyX | copresence | 0.00 | 0.78 |
| 9Pantoea | cdaR | copresence | 0.01 | 0.69 |
| 9Pantoea | dsrA | copresence | 0.01 | 0.69 |
| 9Pantoea | lig | copresence | 0.00 | 0.74 |
