# Supplementary material for: Soil metabolomics and bacterial functional traits revealed the responses of rhizosphere soil bacterial community to long-term continuous cropping of Tibetan barley
Source: PeerJ. 2022 Apr 7;10:e13254. doi: 10.7717/peerj.13254 (PMC8995024; doi:10.7717/peerj.13254)
Supplement: Table S8 [file peerj-10-13254-s016.docx]

**Table S8.** The abundance and detailed information of each metabolite, and the correlation along with enhanced cropping years by Spearman’ correlation test.

| Metabolites | CCY02 | CCY05 | CCY10 | Correlation with enhanced years | kingdom | super_class | class | sub_class |
| --- | --- | --- | --- | --- | --- | --- | --- | --- |
| [2-hydroxy-5-(7-hydroxy-4-oxo-4H-chromen-2-yl)phenyl]oxidanesulfonic acid | 1.96 | 1.94 | 1.78 | DOWN | Organic compounds | Phenylpropanoids and polyketides | Flavonoids | Flavones |
| 13-Hydroxy-9-methoxy-10-oxo-11-octadecenoic acid | 1.79 | 1.77 | 1.40 | DOWN | Organic compounds | Lipids and lipid-like molecules | Fatty Acyls | Lineolic acids and derivatives |
| 3-[4-hydroxy-3-(4-hydroxy-3-methylbut-2-en-1-yl)phenyl]prop-2-enoic acid | 1.89 | 1.50 | 1.40 | DOWN | Organic compounds | Phenylpropanoids and polyketides | Cinnamic acids and derivatives | Hydroxycinnamic acids and derivatives |
| 3-Methyl-3-butenyl apiosyl-(1->6)-glucoside | 3.48 | 3.48 | 3.36 | DOWN | Organic compounds | Lipids and lipid-like molecules | Fatty Acyls | Fatty acyl glycosides |
| 4-Hydroxyandrostenedione glucuronide | 1.54 | 1.06 | 0.79 | DOWN | Organic compounds | Lipids and lipid-like molecules | Steroids and steroid derivatives | Cholestane steroids |
| 4-Nitrophenol | 2.55 | 2.41 | 2.35 | DOWN | Organic compounds | Benzenoids | Phenols | Nitrophenols |
| 5-Heptyltetrahydro-2-oxo-3-furancarboxylic acid | 2.57 | 2.28 | 2.23 | DOWN | Organic compounds | Organoheterocyclic compounds | Lactones | Gamma butyrolactones |
| 6-allyl-8b-Carboxy-ergoline | 1.65 | 0.73 | 0.64 | DOWN | Organic compounds | Alkaloids and derivatives | Ergoline and derivatives | Lysergic acids and derivatives |
| 6''-Malonylcosmosiin | 2.92 | 2.66 | 2.63 | DOWN | Organic compounds | Phenylpropanoids and polyketides | Flavonoids | Flavonoid glycosides |
| 6-Methoxyluteolin 7-glucuronide | 2.39 | 2.14 | 2.08 | DOWN | Organic compounds | Phenylpropanoids and polyketides | Flavonoids | Flavonoid glycosides |
| 6-Methoxy-pyridine-3-carboxylic acid | 1.89 | 1.69 | 1.49 | DOWN | Organic compounds | Organoheterocyclic compounds | Pyridines and derivatives | Pyridinecarboxylic acids and derivatives |
| Alpha-CEHC | 2.99 | 2.97 | 2.47 | DOWN | Organic compounds | Organoheterocyclic compounds | Benzopyrans | 1-benzopyrans |
| Artonin Q | 2.06 | 1.60 | 1.46 | DOWN | Organic compounds | Organoheterocyclic compounds | Benzopyrans | 1-benzopyrans |
| Avenestergenin A1 | 2.44 | 2.37 | 2.19 | DOWN | Organic compounds | Lipids and lipid-like molecules | Prenol lipids | Triterpenoids |
| Capsicum annuum Fluorescent chlorophyll catabolite | 1.85 | 1.72 | 1.31 | DOWN | Organic compounds | Organoheterocyclic compounds | Tetrapyrroles and derivatives | Not Available |
| Clausarinol | 3.86 | 3.76 | 3.74 | DOWN | Organic compounds | Phenylpropanoids and polyketides | Coumarins and derivatives | Pyranocoumarins |
| Corticrocin | 3.08 | 2.68 | 2.68 | DOWN | Organic compounds | Lipids and lipid-like molecules | Fatty Acyls | Fatty acids and conjugates |
| Cyclopassifloic acid B | 3.02 | 2.92 | 2.53 | DOWN | Organic compounds | Lipids and lipid-like molecules | Steroids and steroid derivatives | Cycloartanols and derivatives |
| Diisobutyl phthalate | 2.19 | 2.10 | 2.00 | DOWN | Organic compounds | Benzenoids | Benzene and substituted derivatives | Benzoic acids and derivatives |
| Elenaic acid | 1.52 | 1.29 | 0.94 | DOWN | Organic compounds | Organic acids and derivatives | Carboxylic acids and derivatives | Dicarboxylic acids and derivatives |
| Formylfusarochromanone | 2.17 | 1.94 | 1.63 | DOWN | Organic compounds | Organoheterocyclic compounds | Benzopyrans | 1-benzopyrans |
| Furanofukinin | 2.93 | 2.79 | 2.59 | DOWN | Organic compounds | Lipids and lipid-like molecules | Prenol lipids | Sesquiterpenoids |
| Genipinic acid | 3.06 | 2.93 | 2.79 | DOWN | Organic compounds | Organic acids and derivatives | Carboxylic acids and derivatives | Dicarboxylic acids and derivatives |
| Genistein 4'-rhamnoside | 1.00 | 0.93 | 0.38 | DOWN | Organic compounds | Phenylpropanoids and polyketides | Isoflavonoids | Isoflavonoid O-glycosides |
| Glaucarubolone 15-O-beta-D-glucopyranoside | 2.52 | 2.41 | 2.30 | DOWN | Organic compounds | Lipids and lipid-like molecules | Prenol lipids | Terpene lactones |
| Glutamylvaline | 2.59 | 2.56 | 2.30 | DOWN | Organic compounds | Organic acids and derivatives | Carboxylic acids and derivatives | Amino acids, peptides, and analogues |
| Hematoporphyrin IX | 1.86 | 1.78 | 1.51 | DOWN | Organic compounds | Organoheterocyclic compounds | Tetrapyrroles and derivatives | Porphyrins |
| Isorhamnetin 3-(6''-malonylglucoside) | 1.62 | 1.10 | 1.01 | DOWN | Organic compounds | Phenylpropanoids and polyketides | Flavonoids | Flavonoid glycosides |
| Kamahine C | 2.49 | 2.15 | 1.99 | DOWN | Organic compounds | Organic oxygen compounds | Organooxygen compounds | Ethers |
| L-alpha-Amino-5-oxo-2(5H)-isoxazolepropanoic acid | 1.97 | 1.73 | 1.39 | DOWN | Organic compounds | Organic acids and derivatives | Carboxylic acids and derivatives | Amino acids, peptides, and analogues |
| Methionyl-Proline | 1.57 | 1.53 | 1.31 | DOWN | Organic compounds | Organic acids and derivatives | Carboxylic acids and derivatives | Amino acids, peptides, and analogues |
| Methyl 4-pentenoate | 2.06 | 1.93 | 1.73 | DOWN | Organic compounds | Lipids and lipid-like molecules | Fatty Acyls | Fatty acid esters |
| Monomenthyl succinate | 1.38 | 0.95 | 0.82 | DOWN | Organic compounds | Lipids and lipid-like molecules | Prenol lipids | Monoterpenoids |
| Mytilin A | 2.00 | 1.79 | 1.65 | DOWN | Organic compounds | Organic acids and derivatives | Carboxylic acids and derivatives | Amino acids, peptides, and analogues |
| N-(4-aminobutyl)-3-(4-hydroxyphenyl)propanimidic acid | 1.77 | 1.58 | 1.44 | DOWN | Organic compounds | Benzenoids | Phenols | 1-hydroxy-2-unsubstituted benzenoids |
| Noradrenochrome | 1.79 | 1.70 | 1.61 | DOWN | Organic compounds | Organoheterocyclic compounds | Indoles and derivatives | Not Available |
| Pentigetide | 2.60 | 2.43 | 2.35 | DOWN | Organic compounds | Phenylpropanoids and polyketides | Isoflavonoids | Hydroxyisoflavonoids |
| Phenylpyruvic acid | 1.48 | 1.12 | 1.04 | DOWN | Organic compounds | Benzenoids | Benzene and substituted derivatives | Phenylpyruvic acid derivatives |
| Sphinganine | 4.51 | 4.46 | 4.39 | DOWN | Organic compounds | Organic nitrogen compounds | Organonitrogen compounds | Amines |
| Thioguanosine 5'-diphosphate | 1.79 | 1.53 | 0.67 | DOWN | Organic compounds | Organoheterocyclic compounds | Benzazepines | Dibenzazepines |
| Tridecanol | 3.37 | 3.28 | 3.27 | DOWN | Organic compounds | Lipids and lipid-like molecules | Fatty Acyls | Fatty alcohols |
| Valtrate | 1.27 | 1.07 | 0.54 | DOWN | Organic compounds | Lipids and lipid-like molecules | Prenol lipids | Monoterpenoids |
| Wampetin | 2.27 | 2.01 | 1.90 | DOWN | Organic compounds | Phenylpropanoids and polyketides | Coumarins and derivatives | Furanocoumarins |
| (R)-2-Hydroxysterculic acid | 0.65 | 1.33 | 1.80 | UP | Organic compounds | Lipids and lipid-like molecules | Fatty Acyls | Fatty alcohols |
| (Z)-3-Oxo-2-(2-pentenyl)-1-cyclopenteneacetic acid | 0.54 | 1.14 | 2.25 | UP | Organic compounds | Organic oxygen compounds | Organooxygen compounds | Carbonyl compounds |
| 1-Hydroxy-10-methylacridone | 0.80 | 1.68 | 1.71 | UP | Organic compounds | Organoheterocyclic compounds | Quinolines and derivatives | Benzoquinolines |
| 1-Hydroxy-1-phenyl-3-octadecanone | 1.94 | 2.34 | 2.74 | UP | Organic compounds | Lipids and lipid-like molecules | Fatty Acyls | Fatty alcohols |
| 1-Linoleoylglycerophosphocholine | 4.04 | 4.31 | 4.33 | UP | Organic compounds | Lipids and lipid-like molecules | Glycerophospholipids | Glycerophosphocholines |
| 2-Hydroxy-6-tridecylbenzoic acid | 2.48 | 2.67 | 2.98 | UP | Organic compounds | Benzenoids | Benzene and substituted derivatives | Benzoic acids and derivatives |
| 2-Hydroxyestradiol | 1.36 | 1.80 | 2.07 | UP | Organic compounds | Lipids and lipid-like molecules | Steroids and steroid derivatives | Estrane steroids |
| 2-Nonenoic acid gamma-lactone | 1.03 | 1.53 | 1.61 | UP | Organic compounds | Organoheterocyclic compounds | Dihydrofurans | Furanones |
| 4-(3-hydroxybutyl)-2-methoxyphenol | 1.84 | 2.10 | 2.15 | UP | Organic compounds | Benzenoids | Phenols | Methoxyphenols |
| 4-Deoxyphysalolactone | 1.68 | 1.70 | 1.96 | UP | Organic compounds | Lipids and lipid-like molecules | Steroids and steroid derivatives | Steroid lactones |
| 4-Ethyl-2-hydroxy-3-methyl-2-cyclopenten-1-one | 1.53 | 1.70 | 1.92 | UP | Organic compounds | Organic oxygen compounds | Organooxygen compounds | Carbonyl compounds |
| 4-Ethyl-5-pentyloxazole | 0.65 | 0.73 | 1.67 | UP | Organic compounds | Organoheterocyclic compounds | Azoles | Oxazoles |
| 4-Hydroxy-5-(phenyl)-valeric acid-O-glucuronide | 2.01 | 2.11 | 2.13 | UP | Organic compounds | Organic oxygen compounds | Organooxygen compounds | Carbohydrates and carbohydrate conjugates |
| 4-Hydroxyproline galactoside | 1.45 | 1.77 | 2.13 | UP | Organic compounds | Lipids and lipid-like molecules | Fatty Acyls | Fatty acyl glycosides |
| 4-Hydroxyvalsartan | 1.29 | 1.35 | 2.07 | UP | Organic compounds | Organic acids and derivatives | Carboxylic acids and derivatives | Amino acids, peptides, and analogues |
| 4'-O-Methylkanzonol W | 0.46 | 0.57 | 2.10 | UP | Organic compounds | Phenylpropanoids and polyketides | Isoflavonoids | Pyranoisoflavonoids |
| 5-Hexyltetrahydro-2-furanoctanoic acid | 1.76 | 2.27 | 2.33 | UP | Organic compounds | Lipids and lipid-like molecules | Fatty Acyls | Eicosanoids |
| 5-Hydroxyindoleacetic acid | 1.26 | 1.52 | 1.83 | UP | Organic compounds | Organoheterocyclic compounds | Indoles and derivatives | Indolyl carboxylic acids and derivatives |
| 6alpha-Hydroxyphaseollin | 0.56 | 0.77 | 1.77 | UP | Organic compounds | Phenylpropanoids and polyketides | Isoflavonoids | Furanoisoflavonoids |
| 6-Ketomyristic acid | 1.77 | 2.09 | 2.33 | UP | Organic compounds | Lipids and lipid-like molecules | Fatty Acyls | Fatty acids and conjugates |
| 8-Ocimenyl acetate | 0.18 | 0.57 | 2.50 | UP | Organic compounds | Lipids and lipid-like molecules | Fatty Acyls | Fatty alcohol esters |
| 9(S)-HPODE | 2.09 | 2.46 | 2.84 | UP | Organic compounds | Lipids and lipid-like molecules | Fatty Acyls | Lineolic acids and derivatives |
| Abscisic alcohol 11-glucoside | 0.63 | 1.01 | 1.92 | UP | Organic compounds | Lipids and lipid-like molecules | Prenol lipids | Terpene glycosides |
| Adenine | 1.68 | 2.03 | 2.43 | UP | Organic compounds | Organoheterocyclic compounds | Imidazopyrimidines | Purines and purine derivatives |
| Alanyltryptophan | 2.03 | 2.53 | 2.62 | UP | Organic compounds | Organic acids and derivatives | Carboxylic acids and derivatives | Amino acids, peptides, and analogues |
| Anhydrocinnzeylanol | 0.87 | 0.90 | 2.25 | UP | Organic compounds | Lipids and lipid-like molecules | Prenol lipids | Terpene lactones |
| Arginyl-Histidine | 2.56 | 2.83 | 3.01 | UP | Organic compounds | Organic acids and derivatives | Carboxylic acids and derivatives | Amino acids, peptides, and analogues |
| Artabsinolide D | 0.86 | 1.24 | 1.58 | UP | Organic compounds | Organoheterocyclic compounds | Lactones | Gamma butyrolactones |
| Auberganol | 1.55 | 1.96 | 2.10 | UP | Organic compounds | Lipids and lipid-like molecules | Prenol lipids | Sesquiterpenoids |
| Avenoleic acid | 1.78 | 2.02 | 2.28 | UP | Organic compounds | Lipids and lipid-like molecules | Fatty Acyls | Lineolic acids and derivatives |
| Bakkenolide B | 2.57 | 2.65 | 2.81 | UP | Organic compounds | Lipids and lipid-like molecules | Prenol lipids | Terpene lactones |
| Capsianoside I | 0.44 | 0.49 | 2.31 | UP | Organic compounds | Lipids and lipid-like molecules | Fatty Acyls | Fatty acyl glycosides |
| Celastrol | 2.54 | 2.76 | 2.83 | UP | Organic compounds | Lipids and lipid-like molecules | Prenol lipids | Triterpenoids |
| Choline | 2.38 | 2.45 | 2.74 | UP | Organic compounds | Organic nitrogen compounds | Organonitrogen compounds | Quaternary ammonium salts |
| Chrysaloin | 1.85 | 1.86 | 2.04 | UP | Organic compounds | Benzenoids | Anthracenes | Not Available |
| Cibaric acid | 0.94 | 1.27 | 1.70 | UP | Organic compounds | Lipids and lipid-like molecules | Fatty Acyls | Lineolic acids and derivatives |
| Cinnamoside | 1.79 | 2.28 | 2.38 | UP | Organic compounds | Lipids and lipid-like molecules | Prenol lipids | Terpene glycosides |
| Copalic acid | 1.89 | 2.40 | 2.72 | UP | Organic compounds | Lipids and lipid-like molecules | Prenol lipids | Diterpenoids |
| Creatinine | 0.00 | 0.00 | 0.00 | UP | Organic compounds | Organic acids and derivatives | Carboxylic acids and derivatives | Amino acids, peptides, and analogues |
| Cucurbitacin C | 1.64 | 2.14 | 2.25 | UP | Organic compounds | Lipids and lipid-like molecules | Steroids and steroid derivatives | Cucurbitacins |
| Cytosine | 1.40 | 1.90 | 2.08 | UP | Organic compounds | Organoheterocyclic compounds | Diazines | Pyrimidines and pyrimidine derivatives |
| Deoxycytidine | 1.58 | 1.72 | 1.89 | UP | Organic compounds | Nucleosides, nucleotides, and analogues | Pyrimidine nucleosides | Pyrimidine 2'-deoxyribonucleosides |
| Deoxyinosine | 1.56 | 2.12 | 2.18 | UP | Organic compounds | Nucleosides, nucleotides, and analogues | Purine nucleosides | Purine 2'-deoxyribonucleosides |
| Diosbulbinoside F | 1.80 | 1.95 | 2.38 | UP | Organic compounds | Lipids and lipid-like molecules | Prenol lipids | Terpene glycosides |
| D-Pipecolic acid | 2.09 | 2.27 | 2.66 | UP | Organic compounds | Organic acids and derivatives | Carboxylic acids and derivatives | Amino acids, peptides, and analogues |
| Estradiol | 0.93 | 1.19 | 1.72 | UP | Organic compounds | Lipids and lipid-like molecules | Steroids and steroid derivatives | Estrane steroids |
| Gravacridonediol | 0.36 | 1.24 | 1.60 | UP | Organic compounds | Organoheterocyclic compounds | Quinolines and derivatives | Benzoquinolines |
| Guanine | 1.34 | 1.69 | 2.09 | UP | Organic compounds | Organoheterocyclic compounds | Imidazopyrimidines | Purines and purine derivatives |
| Histidinyl-Arginine | 1.55 | 2.14 | 2.34 | UP | Organic compounds | Organic acids and derivatives | Carboxylic acids and derivatives | Amino acids, peptides, and analogues |
| Hydroxyprolyl-Lysine | 1.37 | 1.41 | 1.62 | UP | Organic compounds | Organic acids and derivatives | Carboxylic acids and derivatives | Amino acids, peptides, and analogues |
| Hypoxanthine | 1.46 | 1.94 | 2.07 | UP | Organic compounds | Organoheterocyclic compounds | Imidazopyrimidines | Purines and purine derivatives |
| Indole-3-carboxylic acid | 1.43 | 1.69 | 1.69 | UP | Organic compounds | Organoheterocyclic compounds | Indoles and derivatives | Indolecarboxylic acids and derivatives |
| Isoliensinine | 0.85 | 0.96 | 2.14 | UP | Organic compounds | Organoheterocyclic compounds | Isoquinolines and derivatives | Benzylisoquinolines |
| Isoputreanine | 0.53 | 0.59 | 0.62 | UP | Organic compounds | Organic acids and derivatives | Carboxylic acids and derivatives | Amino acids, peptides, and analogues |
| Kukoamine B | 1.79 | 1.83 | 2.10 | UP | Organic compounds | Benzenoids | Phenols | Benzenediols |
| Leukotriene E3 | 2.02 | 2.19 | 2.37 | UP | Organic compounds | Lipids and lipid-like molecules | Fatty Acyls | Eicosanoids |
| Linoleoyl Ethanolamide | 3.09 | 3.41 | 3.58 | UP | Organic compounds | Organic nitrogen compounds | Organonitrogen compounds | Amines |
| L-Phenylalanine | 2.45 | 2.87 | 3.12 | UP | Organic compounds | Organic acids and derivatives | Carboxylic acids and derivatives | Amino acids, peptides, and analogues |
| L-trans-alpha-Amino-2-carboxycyclopropaneacetic acid | 2.40 | 2.55 | 2.79 | UP | Organic compounds | Organic acids and derivatives | Carboxylic acids and derivatives | Amino acids, peptides, and analogues |
| L-Tryptophan | 1.65 | 2.01 | 2.15 | UP | Organic compounds | Organoheterocyclic compounds | Indoles and derivatives | Indolyl carboxylic acids and derivatives |
| Lucidenic acid M | 2.21 | 2.49 | 2.59 | UP | Organic compounds | Phenylpropanoids and polyketides | Flavonoids | O-methylated flavonoids |
| LysoPC(15:0) | 3.61 | 3.77 | 3.86 | UP | Organic compounds | Lipids and lipid-like molecules | Glycerophospholipids | Glycerophosphocholines |
| LysoPC(18:1(11Z)) | 4.41 | 4.57 | 4.71 | UP | Organic compounds | Lipids and lipid-like molecules | Glycerophospholipids | Glycerophosphocholines |
| Mangalkanyl glucoside | 1.71 | 1.73 | 2.16 | UP | Organic compounds | Organic oxygen compounds | Organooxygen compounds | Carbohydrates and carbohydrate conjugates |
| Methyl helianthenoate F glucoside | 1.17 | 1.43 | 2.01 | UP | Organic compounds | Lipids and lipid-like molecules | Fatty Acyls | Fatty acyl glycosides |
| MG(0:0/18:1(9Z)/0:0) | 3.04 | 3.15 | 3.35 | UP | Organic compounds | Lipids and lipid-like molecules | Glycerolipids | Monoradylglycerols |
| MG(16:1(9Z)/0:0/0:0) | 3.48 | 3.54 | 4.05 | UP | Organic compounds | Lipids and lipid-like molecules | Glycerolipids | Monoradylglycerols |
| N-(1-Deoxy-b-D-fructopyranosyl) (R)C(S)S-alliin | 1.34 | 2.09 | 2.87 | UP | Organic compounds | Organic acids and derivatives | Carboxylic acids and derivatives | Amino acids, peptides, and analogues |
| N-Benzoylanthranilic acid | 1.16 | 1.26 | 2.25 | UP | Organic compounds | Benzenoids | Benzene and substituted derivatives | Benzoic acids and derivatives |
| Niazirinin | 0.30 | 0.41 | 1.87 | UP | Organic compounds | Organic oxygen compounds | Organooxygen compounds | Carbohydrates and carbohydrate conjugates |
| N-lactoyl-Isoeucine | 2.25 | 2.41 | 2.75 | UP | Organic compounds | Organic acids and derivatives | Keto acids and derivatives | Medium-chain keto acids and derivatives |
| Peonidin 3-rhamnoside | 0.38 | 1.54 | 1.62 | UP | Organic compounds | Phenylpropanoids and polyketides | Flavonoids | Flavonoid glycosides |
| Pimelic acid | 1.62 | 1.99 | 2.15 | UP | Organic compounds | Lipids and lipid-like molecules | Fatty Acyls | Fatty acids and conjugates |
| Prostaglandin F3a | 1.92 | 2.16 | 2.43 | UP | Organic compounds | Lipids and lipid-like molecules | Fatty Acyls | Eicosanoids |
| Prunasin | 1.19 | 2.04 | 2.07 | UP | Organic compounds | Organic oxygen compounds | Organooxygen compounds | Carbohydrates and carbohydrate conjugates |
| Ricinoleic acid | 1.79 | 2.68 | 2.79 | UP | Organic compounds | Lipids and lipid-like molecules | Fatty Acyls | Fatty acids and conjugates |
| Sagittariol | 1.85 | 1.99 | 2.57 | UP | Organic compounds | Lipids and lipid-like molecules | Prenol lipids | Diterpenoids |
| Serylglutamic acid | 1.10 | 2.02 | 2.06 | UP | Organic compounds | Organic acids and derivatives | Carboxylic acids and derivatives | Amino acids, peptides, and analogues |
| Small bacteriocin | 1.28 | 2.12 | 2.28 | UP | Organic compounds | Organic acids and derivatives | Carboxylic acids and derivatives | Amino acids, peptides, and analogues |
| Valyl-Proline | 1.85 | 1.95 | 2.13 | UP | Organic compounds | Organic acids and derivatives | Carboxylic acids and derivatives | Amino acids, peptides, and analogues |
| Vanillin | 1.17 | 1.21 | 1.43 | UP | Organic compounds | Benzenoids | Phenols | Methoxyphenols |
| Xi-4-Hydroxy-4-methyl-2-cyclohexen-1-one | 0.73 | 0.95 | 1.28 | UP | Organic compounds | Organic oxygen compounds | Organooxygen compounds | Carbonyl compounds |
| Yuzu lactone | 0.59 | 1.20 | 2.71 | UP | Organic compounds | Phenylpropanoids and polyketides | Macrolides and analogues | Not Available |
